# Generalized neural closure models with interpretability

**DOI:** 10.1038/s41598-023-35319-w

**Published:** 2023-06-30

**Authors:** Abhinav Gupta, Pierre F. J. Lermusiaux

**Affiliations:** Department of Mechanical Engineering, Center for Computational Science and Engineering, Massachusetts Institute of Technology, Cambridge, MA 02139 USA

**Keywords:** Marine biology, Scientific data, Fluid dynamics, Physical oceanography, Computational science

## Abstract

Improving the predictive capability and computational cost of dynamical models is often at the heart of augmenting computational physics with machine learning (ML). However, most learning results are limited in interpretability and generalization over different computational grid resolutions, initial and boundary conditions, domain geometries, and physical or problem-specific parameters. In the present study, we simultaneously address all these challenges by developing the novel and versatile methodology of unified neural partial delay differential equations. We augment existing/low-fidelity dynamical models directly in their partial differential equation (PDE) forms with both Markovian and non-Markovian neural network (NN) closure parameterizations. The melding of the existing models with NNs in the continuous spatiotemporal space followed by numerical discretization automatically allows for the desired generalizability. The Markovian term is designed to enable extraction of its analytical form and thus provides interpretability. The non-Markovian terms allow accounting for inherently missing time delays needed to represent the real world. Our flexible modeling framework provides full autonomy for the design of the unknown closure terms such as using any linear-, shallow-, or deep-NN architectures, selecting the span of the input function libraries, and using either or both Markovian and non-Markovian closure terms, all in accord with prior knowledge. We obtain adjoint PDEs in the continuous form, thus enabling direct implementation across differentiable and non-differentiable computational physics codes, different ML frameworks, and treatment of nonuniformly-spaced spatiotemporal training data. We demonstrate the new *generalized neural closure models* (*g*nCMs) framework using four sets of experiments based on advecting nonlinear waves, shocks, and ocean acidification models. Our learned *g*nCMs discover missing physics, find leading numerical error terms, discriminate among candidate functional forms in an interpretable fashion, achieve generalization, and compensate for the lack of complexity in simpler models. Finally, we analyze the computational advantages of our new framework.

## Introduction

The field of Scientific Machine Learning (SciML^1^) is burgeoning with innovative methods that combine machine learning with existing scientifically-derived differential equation models and computational physics schemes. This is in part because many realistic dynamical models are complex, and often truncated, coarsened, or aggregated due to computational cost constraints. Machine learning (ML) is then used to learn and represent the neglected and unresolved terms in a data-driven fashion^2–9^. Such techniques that express the missing dynamics as functions of modeled state variables and parameters are referred to as closure models. Most ML closure models (and SciML results in general) are however often limited both in interpretability as black-box ML models and in generalization over different computational grid resolutions, initial conditions, boundary conditions, domain geometries, and physical or problem-specific parameters. Addressing the challenges of interpretability and generalization is imperative to justify the costs of training the SciML models using data sets obtained from expensive measurements or generated by solving the complex dynamical models in the first place. The goal of the present study is to simultaneously address these challenges and learn closure models which are both generalizable and interpretable.

The need for closure modeling arises for a variety of reasons, ranging from computational cost considerations, preference for simpler models over complex ones due to overparameterization, or lack of scientific understanding of processes and variables involved in the system of interest. The simpler or the known model is often referred to as a low-fidelity model, while the complex counterpart in either models or observations is then referred to as the high-fidelity model, reality, or real-world data. Low-fidelity models can be categorized into three categories: (i) Reduced-order models, in which the original high-dimensional dynamical system is projected and solved in a reduced space. While it is computationally cheaper to solve the low-dimensional system, these models can quickly accumulate errors due to the missing interactions with the truncated dimensions^10–12^; (ii) Coarse-resolution models, in which we only resolve the scales of interest. In these cases, the neglected and unresolved scales, along with their interactions with the resolved ones, can lead to unintended or unacceptable effects at global scales^13–17^; (iii) Simplistic or speculative models, in which an incomplete representation or understanding of processes and interactions occurs, and thus uncertainty in the model formulations and even in the relevant state variables themselves. This can lead to a gross or incorrect approximation of the real-world phenomena^18–22^.

In^2^, neural closure models (nCMs) are developed for low-fidelity models using neural delay differential equations (nDDEs) and data from high-fidelity simulations. The need for time delays in closure parameterizations is rooted in the presence of inherent delays in real-world systems^23,24^ and theoretically justifed by the Mori–Zwanzig formulation^25–28^. Using nDDEs for closure modeling has a number of advantages. They allow for the use of smaller architectures and account for the accumulation of numerical time-stepping error in the presence of neural networks (NNs) during training. Additionally, nDDEs are agnostic to the time-integration scheme, handle unevenly-spaced training data, and have good performance over prediction periods much longer than the training or validation periods. However, there are other highly-desirable properties, as mentioned above. Fundamental questions for neural closures include: Can they be interpretable and lead to analytical expressions? Can they achieve generalization over many conditions and variables, as physics-based models do? How can they be combined seamlessly with classic numerical schemes? A number of recent approaches have aimed to address such questions, however, challenges remain especially for partial differential equations (PDEs). This is often because NNs are used with the discretized ordinary differential equation (ODE) form of the corresponding PDEs, which makes it inherently difficult to generalize to changes in boundary conditions, domain geometry, and computational grid. Recently, a few studies have taken steps at addressing these drawbacks. Sirignano et al.^8^ augment the underlying PDE with a neural network, however, they only learn a Markovian closure. The inputs to the neural network include the state, its spatial derivatives, and a fixed number of neighboring grid points. They also provide an accompanying discrete adjoint PDE for efficient training. Saha et al.^9^ use a radial-basis-functions-based collocation method to allow for mesh-free embedding of NNs. However, the resulting NNs also only learn a Markovian closure, do not account for the accumulation of time-integration errors, and lack interpretability.

In the present study, we develop the unified neural partial delay differential equations (nPDDEs) that augment existing/low-fidelity models in their PDE forms with both Markovian and non-Markovian closures parameterized with deep-NNs. The neural closure terms then contain instantaneous and delayed contributions. Their inputs consist of the modeled states, their spatial derivatives, combinations of derivatives, and any other problem-specific variables and parameters. The melding of the low-fidelity model and deep-NNs in the continuous spatiotemporal space automatically allows for generalizability to computational grid resolution, boundary conditions, and initial conditions. By design, the closure terms can also provide analytical expressions of the missing terms, thus leading to interpretability. The resulting nPDDEs are discretized using any numerical method relevant to the dynamical system studied. Further, we provide adjoint PDE derivations in the continuous form, thus allowing one to implement across differentiable and non-differentiable computational physics codes, and also different machine learning frameworks. All our derivations and implementations are done considering deep-NN architectures, thus automatically encompassing linear- and shallow-NNs, and providing the user or subject-matter-expert user with the flexibility of choosing the architectural complexity in accord with the prior information available. We refer to the new methodology as *generalized* neural closure models (*g*nCM). Through a series of experiments, we demonstrate the flexibility of *g*nCMs to learn closures either in an interpretable fashion, black-box fashion, or both simultaneously, using the prior scientific knowledge about the problem at hand. The *g*nCMs can eliminate erroneous and redundant input terms, or combine them to achieve increased accuracy. We also demonstrate the generalizability of our learned closures to changes in physical parameters, grid resolution, initial conditions, and boundary conditions. Our first class of simulation experiments uses nonlinear waves and advecting shocks problems governed by the KdV-Burgers and classic Burgers PDEs. Our learned *g*nCM finds missing terms, discovers the leading truncation error, and a correction to the non-linear advection term. We find that training on data corresponding to just a few combinations of grid resolution and Reynolds number is sufficient to ensure that the learned closures are generalizable over a range of grid resolution and Reynolds number combinations, initial and boundary conditions, and also outperform the popular Smagorinsky subgrid-scale closure model. Our second class of experiments is based on ocean acidification models, where we learn the functional form of biological processes and compensate for the lack of complexity in simpler models obtained by aggregation of components and other simplifications of processes and parameterizations. Finally, we comment on the computational advantages of our new *g*nCM framework.

In what follows, we first develop the “Theory and methodology” for the gnCMs. “Application results and discussion” showcases the generalization and interpretability properties of the gnCMs in experiments with nonlinear waves, advecting shocks, and ocean acidification, and discusses computational advantages. Finally, “Conclusions” are provided.

## Theory and methodology

The functional form of closure models representing missing dynamics can be derived by the Mori–Zwanzig formulation^25–28^, which proves it to be dependent on the time-lagged state dynamics. Many systems are modeled assuming smooth fields of state variables governed by advection-diffusion-reaction PDEs. Such PDEs implicitly assume that local information between state variables is exchanged instantaneously at any spatial location. In reality, however, time delays occur for several reasons. First, reactions or changes in populations have non-negligible time scales. Such time delays are captured in more complex models by modeling intermediate state variables. The time response of lower-complexity models can thus approximate that of high-complexity models by explicitly introducing delays^23,24^. Second, time delays arise due to missing subgrid-scale processes and/or truncated modes in reduced-order models. For all of these reasons, memory-based terms and thus non-Markovian closure terms are needed to augment low-fidelity models^2^.

In general, low-fidelity models are also outright missing Markovian terms due to truncation, coarse resolution, or incomplete and uncertain functional forms of some of the model terms. We will therefore use both Markovian and non-Markovian terms to close low-fidelity models in their PDE forms. This leads to partial delay differential equations (PDDEs) that are widely used in ecology, control theory, biology, and climate dynamics, to name a few application areas^29^.

In this study, the Markovian and non-Markovian closure terms will be modeled using deep-NNs. To achieve full interpretability from the learned weights of the NNs of the closure models, we at times consider single-layer linear-NNs. Closure terms in general depend on the state variables, their spatial derivatives, and combinations of these belonging to a function library. As the presence of discrete delays can be seen as a special case of distributed delays, the non-Markovian term is assumed to contain distributed delays and have a maximum finite time-delay ($\tau$). Given a continuous state vector comprising of $N_s$ different states, $u(x, t): {\mathbb {R}} \times [0, T] \rightarrow {\mathbb {R}}^{N_s}$, we thus consider a dynamical system belonging to domain $\Omega$ of the following form,

1 $$\begin{aligned} \begin{aligned} \frac{\partial u(x, t)}{\partial t}&= \underbrace{{\mathcal {L}}\left( u(x, t), \frac{\partial u(x, t)}{\partial x}, \frac{\partial ^2 u(x, t)}{\partial x^2},..., x, t; \nu \right) }_{Low-Fidelity~/~Known~Model} \\&+ \underbrace{{\mathcal {F}}_{NN}\left( u(x, t), \frac{\partial u(x, t)}{\partial x}, \frac{\partial ^2 u(x, t)}{\partial x^2},..., x, t; \phi \right) }_{Markovian~Closure~Term} \\&+ \underbrace{\int _{t-\tau }^t {\mathcal {D}}_{NN}\left( u(x, s), \frac{\partial u(x, s)}{\partial x}, \frac{\partial ^2 u(x, s)}{\partial x^2},..., x, s; \theta \right) ds}_{Non-Markovian~Closure~Term}, \quad x\in \Omega , \; t \ge 0\,, \\ u(x, t)&= h(x, t), \; -\tau \le t \le 0 \quad \text {and} \quad {\mathcal {B}}(u(x, t)) = g(x, t), \; x \in \partial \Omega , \; t \ge 0 , \end{aligned} \end{aligned}$$

where ${\mathcal {L}}$, ${\mathcal {F}}_{NN}$, and ${\mathcal {D}}_{NN}$ are nonlinear functions parameterized with $\nu$, $\phi$, and $\theta$, respectively. $\nu$ are problem-specific parameters associated with the physical/biological/chemical phenomenon of interest, while $\phi$ and $\theta$ are the NN weights. When compared to PDEs, PDDEs require a history function ($h(x, t)\,, \; -\tau \le t \le 0$) for their initialization at $t=0$. The operator ${\mathcal {B}}$ represents appropriate boundary conditions such as Dirichlet, Neumann, etc. which are needed to solve the system uniquely. Furthermore, for ease of notation, we have assumed a one-dimensional (1D) domain, however, the method directly extends to 2D and 3D domains.

### Neural partial delay differential equations

We now obtain ML schemes that learn PDDEs parameterized using deep-NNs. They are referred to as *neural* partial delay differential equations (*n*PDDEs). Without loss of generality, and for brevity, we limit ourselves to *n*PDDEs with only a Markovian term and a non-Markovian term with distributed delays. The low-fidelity model can be considered to be absorbed in the Markovian closure term. Hence, the *n*PDDE is of the form,

2 $$\begin{aligned} \begin{aligned} \frac{\partial u(x, t)}{\partial t}&= {\mathcal {F}}_{NN}\left( u(x, t), \frac{\partial u(x, t)}{\partial x}, \frac{\partial ^2 u(x, t)}{\partial x^2},..., \frac{\partial ^d u(x, t)}{\partial x^d}, x, t; \phi \right) \\&+ \int _{t-\tau }^t {\mathcal {D}}_{NN}\left( u(x, s), \frac{\partial u(x, s)}{\partial x}, \frac{\partial ^2 u(x, s)}{\partial x^2},..., \frac{\partial ^d u(x, s)}{\partial x^d}, x, s; \theta \right) ds , \\&\quad x\in \Omega , \; t \ge 0, \\ u(x, t)&= h(x, t), \; -\tau \le t \le 0 \quad \text {and} \quad {\mathcal {B}}(u(x, t)) = g(x, t) \, \quad x \in \partial \Omega , \; t \ge 0 . \end{aligned} \end{aligned}$$

The two deep-NNs, instantaneous ${\mathcal {F}}_{NN}(\bullet ; \phi )$ and delayed ${\mathcal {D}}_{NN}(\bullet ; \theta )$, remain parameterized by $\phi$ and $\theta$, and for generality, they are considered to be functions of an arbitrary number of spatial derivatives, with the highest order defined by $d \in {\mathbb {Z}}^+$. We can rewrite Eq. (2) as an equivalent system of coupled PDDEs with discrete delays,

3 $$\begin{aligned} \begin{aligned} \frac{\partial u(x, t)}{\partial t}&= {\mathcal {F}}_{NN}\left( u(x, t), \frac{\partial u(x, t)}{\partial x}, \frac{\partial ^2 u(x, t)}{\partial x^2},..., \frac{\partial ^d u(x, t)}{\partial x^d}, x, t; \phi \right) + y(x, t) , \\&\quad x\in \Omega , \; t \ge 0 , \\ \frac{\partial y(x, t)}{\partial t}&= {\mathcal {D}}_{NN}\left( u(x, t), \frac{\partial u(x, t)}{\partial x}, \frac{\partial ^2 u(x, t)}{\partial x^2},..., \frac{\partial ^d u(x, t)}{\partial x^d}, x, t; \theta \right) \\&- {\mathcal {D}}_{NN}\left( u(x, t-\tau ), \frac{\partial u(x, t-\tau )}{\partial x}, \frac{\partial ^2 u(x, t-\tau )}{\partial x^2},..., \frac{\partial ^d u(x, t-\tau )}{\partial x^d}, x, t-\tau ; \theta \right) , \\&\quad x\in \Omega , \; t \ge 0 ,\\ u(x, t)&= h(x, t), \; -\tau \le t \le 0 \quad \text {and} \quad {\mathcal {B}}(u(x, t)) = g(x, t), \; x \in \partial \Omega , \; t \ge 0 , \\ y(x, 0)&= \int _{-\tau }^0 {\mathcal {D}}_{NN}\left( h(x, s), \frac{\partial h(x, s)}{\partial x}, \frac{\partial ^2 h(x, s)}{\partial x^2},..., \frac{\partial ^d h(x, s)}{\partial x^d}, x, s; \theta \right) ds . \end{aligned} \end{aligned}$$

Let us assume that high-fidelity data is available at *M* discrete times, $T_1<...<T_M \le T$, and at $N(T_i)$ spatial locations ($x_{k}^{T_i} \in \Omega , \forall k \in {1,..., N(T_i)}$) for each of the times. Thus, we define the scalar loss function as, $L = \frac{1}{M} \sum _{i=1}^{M} \frac{1}{N(T_i)}\sum _{k=1}^{N(T_i)} l(u(x^{T_i}_k, T_i)) \equiv \int _0^T \frac{1}{M} \sum _{i=1}^{M} \int _{\Omega } \frac{1}{N(T_i)}\sum _{k=1}^{N(T_i)} l(u(x, t))\delta (x - x^{T_i}_k) \delta (t - T_i)dxdt \equiv \int _0^T \frac{1}{M} \sum _{i=1}^{M} \frac{1}{|\Omega |} \int _{\Omega } {\hat{l}}(u(x, t))\delta (t - T_i) dxdt$, where $l(\bullet )$ are scalar loss functions such as mean-absolute-error (MAE), and $\delta (\bullet )$ is the Kronecker delta function. In order to derive the adjoint PDEs, we start with the Lagrangian corresponding to the above system,

4 $$\begin{aligned} \begin{aligned} {\mathbb {L}}&= L(u(x, t)) + \int _0^T \int _{\Omega }\lambda ^T(x, t) \left( \partial _t u(x, t) - {\mathcal {F}}_{NN}(\bullet , t; \phi ) - y(x, t)\right) dxdt \\&+ \int _0^T \int _{\Omega }\mu ^T(x, t) \left( \partial _t y(x, t) - {\mathcal {D}}_{NN}(\bullet , t;\theta ) + {\mathcal {D}}_{NN}(\bullet , t-\tau ; \theta )\right) dxdt \\&+ \int _{\Omega } \alpha ^T(x) \left( y(x, 0) - \int _{-\tau }^0 {\mathcal {D}}_{NN}(h(x, t), \partial _x h(x, t), \partial _{x^2} h(x, t),..., \partial _{x^d} h(x, t), x, t; \theta )dt\right) dx , \end{aligned} \end{aligned}$$

where $\lambda (x, t)$, $\mu (x, t)$ and $\alpha (x)$ are the Lagrangian variables. To find the gradients of ${\mathbb {L}}$ w.r.t. $\phi$ and $\theta$, we first solve the following adjoint PDEs (for brevity we denote, $\partial / \partial (\bullet ) \equiv \partial _{(\bullet )}$, and $d / d (\bullet ) \equiv d_{(\bullet )}$),

5 $$\begin{aligned} \begin{aligned} 0&= \frac{1}{M}\frac{1}{|\Omega |} \sum _{k=1}^M \partial _{u(x, t)} {\hat{l}}(u(x, t)) \delta (t - T_k) \\&- \partial _t \lambda ^T(x, t) - \lambda ^T(x, t)\partial _{u(x, t)}{\mathcal {F}}_{NN}(\bullet , t) + \sum _{i=1}^d (-1)^{i+1} \partial _{x^i} \left( \lambda ^T(x, t) \partial _{\partial _{x^i} u(x, t)} {\mathcal {F}}_{NN}(\bullet , t) \right) \\&- \mu ^T(x, t) \partial _{u(x, t)} {\mathcal {D}}_{NN}(\bullet , t; \theta ) + \sum _{i=1}^d (-1)^{i+1} \partial _{x^i} \left( \mu ^T(x, t) \partial _{\partial _{x^i} u(x, t)} {\mathcal {D}}_{NN}(\bullet , t; \theta ) \right) \\&+ \mu ^T(x, t+\tau ) \partial _{u(x, t)} {\mathcal {D}}_{NN}(\bullet , t; \theta ) - \sum _{i=1}^d (-1)^{i+1} \partial _{x^i} \left( \mu ^T(x, t+\tau ) \partial _{\partial _{x^i} u(x, t)} {\mathcal {D}}_{NN}(\bullet , t; \theta ) \right) , \\&\quad x \in \Omega , \; t \in [0, T) ,\\ 0&= -\lambda ^T(x, t) - \partial _t \mu ^T(x, t) , \quad x \in \Omega , \; t \in [0, T) , \end{aligned} \end{aligned}$$

with initial conditions, $\lambda (x, t) =\mu (x, t) = 0, \; t \ge T$. The boundary conditions are derived based on those of the forward PDDE and they satisfy,

6 $$\begin{aligned} \begin{aligned} 0&= \sum _{i = 0}^d \sum _{j=0}^{d-i-1} (-1)^{j+1} \partial _{x^{j}} \left( \lambda ^T(x, t)\partial _{\partial _{x^{j+i+1}} u(x, t)} {\mathcal {F}}_{NN}(\bullet , t)\right) d_{\theta }\partial _{x^{i}}u(x, t) \\&+\sum _{i = 0}^d\sum _{j=0}^{d-i-1} (-1)^{j+1} \partial _{x^{j}} \left( \mu ^T(x, t)\partial _{\partial _{x^{j+i+1}} u(x, t)} {\mathcal {D}}_{NN}(\bullet , t)\right) d_{\theta }\partial _{x^{i}}u(x, t) \\&- \sum _{i = 0}^d\sum _{j=0}^{d-i-1} (-1)^{j+1} \partial _{x^{j}} \left( \mu ^T(x, t+\tau )\partial _{\partial _{x^{j+i+1}} u(x, t)} {\mathcal {D}}_{NN}(\bullet , t)\right) d_{\theta }\partial _{x^{i}}u(x, t) , \\&\quad x \in \partial \Omega , \; t \in [t, T) . \end{aligned} \end{aligned}$$

Details of the derivation of the above adjoint PDEs are in the Supplementary information, Sect. SI-1. After solving for the Lagrangian variables, $\lambda (x, t)$ and $\mu (x, t)$, we compute the required gradients as,

7 $$\begin{aligned} \begin{aligned} d_{\theta }{\mathcal {L}}&= - \int _0^T \int _{\Omega }\mu ^T(x, t) \partial _{\theta }{\mathcal {D}}_{NN}(\bullet , t; \theta ) dxdt + \int _0^T \int _{\Omega }\mu ^T(x, t) \partial _{\theta }{\mathcal {D}}_{NN}(\bullet , t-\tau ; \theta ) dxdt \\&- \int _{\Omega } \mu ^T(x, 0) \int _{-\tau }^0 \partial _{\theta }{\mathcal 
{D}}_{NN}(h(x, 
t), \partial _x h(x, t), 
\partial _{xx} h(x, t), x, t; \theta )dt dx , \\ d_{\phi }{\mathcal {L}}&= - \int _0^T \int _{\Omega }\lambda ^T(x, t) \partial _{\phi }{\mathcal {F}}_{NN}(\bullet , t; \phi ) dxdt . \end{aligned} \end{aligned}$$

Finally, using a stochastic gradient descent algorithm, we find the optimal values of the weights $\phi$ and $\theta$.

### Generalized neural closure models: properties

**Figure 1:**
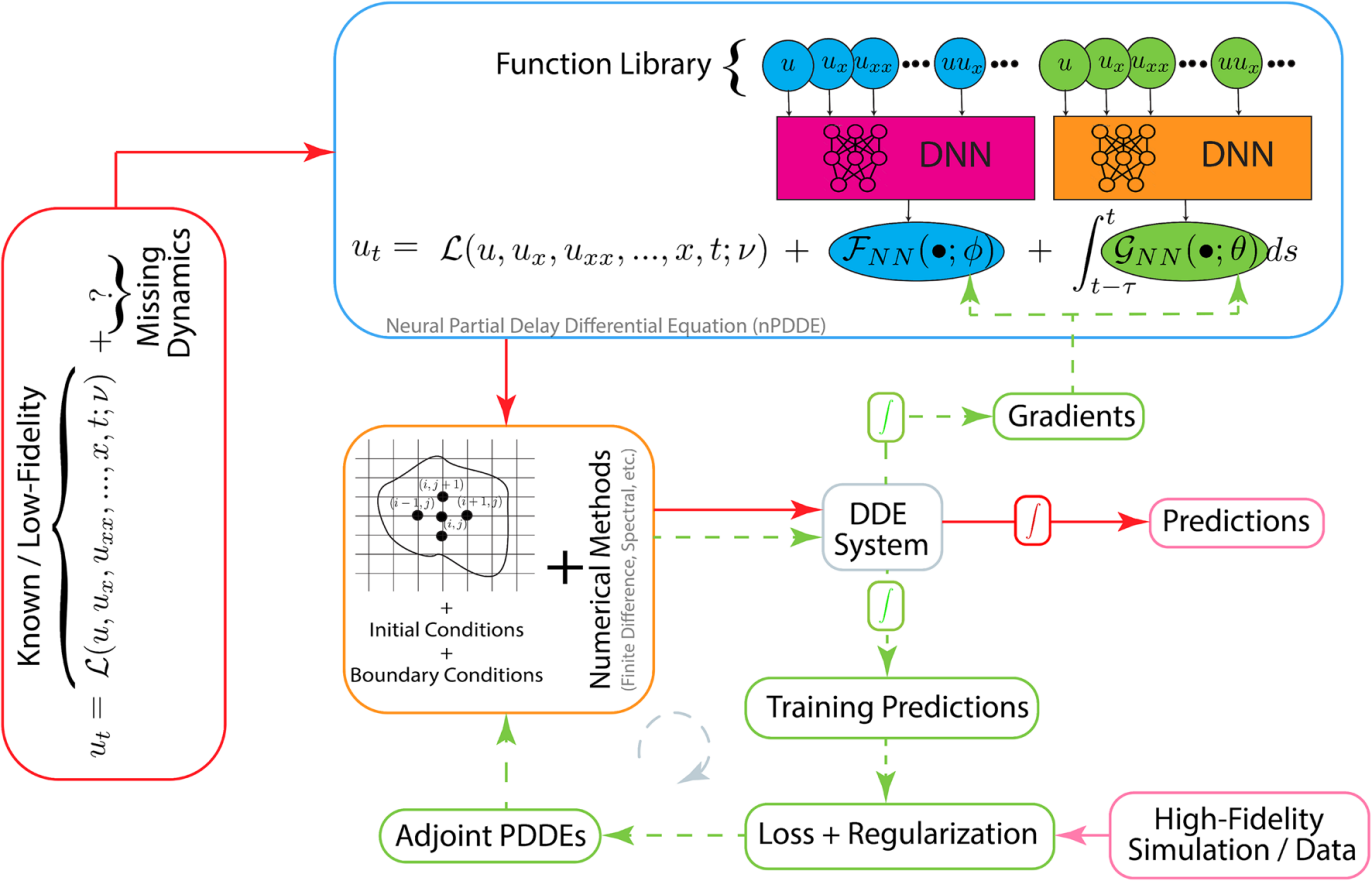
Overview of the *generalized* neural closure models (*g*nCM) framework. The blocks labeled *DNN* represent any deep-neural-network architectures. The block labeled $\int$ symbolizes any time-integration scheme. DDE stands for delay differential equation.

The *g*nCM framework is schematized in Fig. 1. Next, we discuss some of its properties and variations.

*Interpretability* For interpretability—especially for the Markovian closure term—we can use a simple NN architecture with no hidden layers and linear activation. Nonlinearity can still be introduced by having input features that are nonlinear combinations of the states and their derivatives belonging to a function library. The result is a linear combination of these nonlinear input features. Along with this, a $L_1$ regularization on the NN weights and pruning below a threshold helps promote sparsity, thus allowing for redundancy in the input function library. In practice, one can include as many input test functions as computationally efficient and scientifically meaningful, and then adaptively augment and prune this library during the data-driven learning process, similar to that demonstrated in^30^. Although this approach has similarities to SINDy^30–32^, it is significantly different. SINDy requires training data to be rich enough to allow for the computation of temporal and spatial derivatives, and solves a regression problem to discover the governing dynamical system. Some successors of SINDy circumvent the need for calculating spatio-temporal derivatives from training data by utilizing weak forms^33^ and NNs to map coordinates of the problem to the state variable^34^. Our *g*nCM method also does not require using the training data to compute any temporal and spatial derivatives. It further accounts for the accumulation of time-integration errors during training by numerically solving the PDE augmented with the Markovian closure term and its corresponding adjoint PDE. Compared to other model discovery methods, *g*nCM seamlessly incorporates and simultaneously learns a non-Markovian closure term without simplifying assumptions.

The use of an informative function library along with a simple NN architecture with no hidden layers and linear activation is also applicable for the non-Markovian term for enhanced interpretability. In fact, in our derivation in the prior section and framework implementation, we keep the possibility of using any general deep-NN architectures for both Markovian and non-Markovian closure terms. This allows one to introduce an arbitrary amount of nonlinearity, especially in cases when no prior information is available about the functional form of the missing dynamics. The use of deep-NNs comes at the cost of full interpretability. However, even in this case, some insight can be obtained, for example, by examining the weights of the input layer of the learned deep-NN to determine the relative importance of different input features. This is showcased in “Experiments 1b: advecting shock—model discovery and generalization” for the learned non-Markovian closure term.

*Generalizability* The forward model (Eq. 1 or 2) and the adjoint PDEs (Eq. 5) are discretized and integrated using numerical schemes^35^, such as finite differences, finite volumes, collocation methods, etc. This new approach, where we augment the PDEs with the NN-based Markovian and non-Markovian closures first, before numerical discretization, ensures that the burden of generalization over boundary conditions, domain geometry, and computational grid resolution, along with computing the relevant spatial derivatives is handled by the numerical schemes, and not by the learned deep-NNs. This also automatically makes the learning only dependent on local features and affine equivariant, similar to numerical schemes.

*Backpropagation and adjoint equations* With the *adjoint method*, the adjoint PDEs (Eqs. 5 and 6) are solved backward in time, and one would require access to $u(x, t), \forall x \in \Omega , \; 0 \le t \le T$. In the original neural ODEs^36^, the proposed adjoint method forgets the forward-time trajectory $u(x, t), \forall x \in \Omega , \; 0 \le t \le T$; instead, it remembers only the state at the final time, *u*(*x*, *T*), and then solves for *u*(*x*, *t*) in reverse-time along with the adjoint PDEs. This approach is known to suffer from inaccuracies and numerical instabilities^37,38^. Thus, in our current implementation, we create and continuously update an interpolation function using the *u* obtained at every time step as we solve the forward model (Eq. 2). For memory efficiency, one could also use the method of *checkpointing*^37–39^, or the interpolated reverse dynamic method (IRDM)^40^. Along with this, using adaptive time-integration schemes leads to stable and accurate solutions for our forward and adjoint PDEs, especially for stiff dynamical systems^41,42^. The inherent inaccuracies and instabilities of using continuous adjoint equations followed by discretization remain open questions^37,38,41–43^ and well-known issues in data assimilation^44,45^. In this work, we found that the combination of the continuous adjoint method followed by discretization and adaptive time-integration schemes is successful. Another challenge that can occur is the feasibility of derivation of the continuous adjoint PDEs followed by discretization, especially for known realistic (low-fidelity) models that are highly complex and nonlinear. In such cases, the discrete adjoint method, i.e., the approach of deriving the adjoint equations for the discrete forward model might be more useful^46,47^. This makes it easier to utilize the vast array of tools developed by the *Automatic Differentiation* community over the last several decades^48^, specifically, the source-code-transformation (source-to-source) methods^49,50^. Finally, reduced-space adjoints as well as ensemble approaches can be used to estimate gradients^51^.

## Application results and discussion

Using four sets of experiments, we now showcase and evaluate the capabilities of our new closure modeling framework (*g*nCM) in terms of generalizability over grid resolutions, boundary and initial conditions, and problem-specific parameters. We also demonstrate the interpretability of the learned closures within PDEs.

In the first and second sets of experiments, we consider problems based on advecting nonlinear wave and shock PDEs. We find that *g*nCMs can discriminate and discover processes such as dispersion, the leading truncation error term, and a correction to the nonlinear advection term, all in an interpretable fashion with the learned Markovian neural closure. Using both Markovian and non-Markovian neural closure terms, we demonstrate the generalization of the *g*nCM over grid resolution, Reynolds number, and initial and boundary conditions, along with superior performance compared to the popular Smagorinsky closure. In the third and fourth sets of experiments, we consider problems based on coupled physical-biological-carbonate PDEs used to study the threat of ocean acidification. We utilize the *g*nCMs to discriminate and discover the functional form of uncertain terms with interpretability, and to augment a simpler model obtained by aggregation of components and simplifications of processes and parameterizations, such that, it becomes as accurate as a more complex model.

Our training and evaluation protocol is similar to that in^2^. We use performance over the validation period (past the period for which high-fidelity data snapshots are used for training) to fine-tune various training-related hyperparameters. The final evaluation is based on continuous evolution through the training and validation periods, followed by longer-term future predictions. We also compare the learned closure with the known true model. For all the figure, table, and section references prefixed with “SI-”, we direct the reader to the *Supplementary Information*.

### Experiments 1a: nonlinear waves—interpretable model discrimination

In the first set of experiments, we consider advecting nonlinear wave PDEs and discover missing/uncertain physical processes, such as dispersion, in an interpretable fashion, using the learned Markovian neural closure.

*Setup: true model, data generation, and low-fidelity model* Models for advecting shocks and nonlinear waves are the backbone of various physical phenomena. The Korteweg de Vries (KdV)–Burgers PDE is often used to describe weak effects of dispersion, dissipation, and non-linearity in such wave propagation^52^. Here, considering a 1D spatial domain, we select this KdV–Burgers PDE as the high-fidelity model (truth),

8 $$\begin{aligned} \begin{aligned} \frac{\partial u}{\partial t} = -6u\frac{\partial u}{\partial x} - \frac{\partial ^3 u}{\partial x^3} . \end{aligned} \end{aligned}$$

The data is generated from two solitary waves colliding with each other and that are exact solutions of Eq. (8) with initial and boundary conditions given by,

9 $$\begin{aligned} \begin{aligned} u(x, 0)&= 2 \eta _1^2 \text {sech}[\eta _1(x - x_1)] + 2 \eta _2^2 \text {sech}[\eta _2(x - x_2)], \\ u(-L, t)&= 0, \; \frac{\partial u (x, t)}{\partial x} \bigg |_{x = L} = 0, \; \text {and} \; \frac{\partial ^2 u (x, t)}{\partial x^2} \bigg |_{x = L} = 0 , \end{aligned} \end{aligned}$$

where $x_1$ is the location, $2 \eta _1^2$ is the amplitude, and $1/\eta _1$ is the width of the first soliton wave, whereas $x_2$ is the location, $2 \eta _2^2$ is the amplitude, and $1/\eta _2$ is the width of the second soliton wave, initially. The parametric analytical solution of the above system is given by,

10 $$\begin{aligned} \begin{aligned} u(x, t) = \frac{8(\eta _1^2 - \eta _2^2) (\eta _1^2 \cosh {\theta _2} + \eta _2^2 \sinh {\theta _1})}{( (\eta _1 - \eta _2) \cosh (\theta _1 + \theta _2) + (\eta _1 + \eta _2) \cosh (\theta _1 - \theta _2))^2} , \end{aligned} \end{aligned}$$

where $\eta _1 \ge \eta _2$, and $\theta _1$ and $\theta _2$ are given by,

11 $$\begin{aligned} \begin{aligned} \theta _1&= \eta _1(x-x_1 - 4 \eta _1^2 t) , \\ \theta _2&= \eta _1(x-x_2 - 4 \eta _2^2 t) . \end{aligned} \end{aligned}$$

We choose $L=10$, maximum time $T = 1.5$, $\eta _1 = 1.2$, $\eta _2 = 0.8$, $x_1 = -6.0$ and $x_2 = -2.0$.

For the closure learning experiments, we assume we only have prior knowledge about the existence of the advection term and the low-fidelity model is thus,

12 $$\begin{aligned} \begin{aligned} \frac{\partial u}{\partial t} = -u\frac{\partial u}{\partial x} . \end{aligned} \end{aligned}$$

Other effects are unknown and need to be discovered. We assume these unknown effects to be mainly Markovian in nature and that they can be modeled using a linear combination from a library of nonlinear functions comprising terms up to 3rd order spatial derivatives: $\left\{ \frac{\partial ^2 u}{\partial x^2}, \frac{\partial ^3 u}{\partial x^3}, u\frac{\partial u}{\partial x}, u^2 \frac{\partial u}{\partial x}\right\}$. Compared to the true model (Eq. 8), our library contains two superfluous or redundant terms, $\frac{\partial ^2 u}{\partial x^2}$ and $u^2 \frac{\partial u}{\partial x}$. Of course, it does not contain repetitive terms.

**Figure 2:**
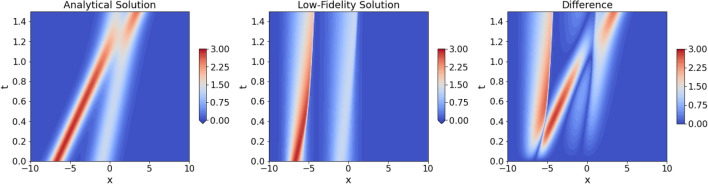
Comparison of the numerical solution of the KdV–Burgers equation with only the advection term (Eq. 12; low-fidelity model; *middle plot*), with the analytical solution corresponding to the equation with stronger advection and 3rd order derivative term (Eqs. 8, 9 and 10; high-fidelity model; left plot). The low-fidelity model is solved on a grid with $N_x=200$ grid points. The absolute difference between the two solutions is provided in the right panel.

*Numerics* All the numerical solutions of low-fidelity model augmented with the *g*nCM are obtained using finite difference schemes. For the advection term, $2^{nd}$ order accurate upwind^53^ is used, while all other spatial terms and derivatives are discretized with $4^{th}$ order accurate central-difference. For time-marching, the *Vode* scheme^54^ with adaptive time-stepping is used. Finally, we employ a fine grid with $N_x = 200$ number of grid points in the $x-$direction in order to keep low discretization and truncation errors. *Comparison LF-HF:* in Fig. 2, we compare the numerical solution of the low-fidelity model (Eq. 12) with the analytical solution of the high-fidelity model (Eqs. 8, 9 and 10). The solutions of the two models have the same initial condition, however, their evolutions are drastically different. With the high-fidelity model, the two solitons interact elastically, i.e., their amplitudes and shapes are unchanged after the interaction, however, they do experience a phase shift in their positions. With the low-fidelity model, however, the two solitons do not even come close to interacting with each other.

*Training: NN architecture, data, and loss function* For the *g*nCM, we only consider the Markovian term with a simple neural network with no hidden layer and only linear activation in the output layer, in-effect equivalent to a linear combination of the inputs. The training data consists of the analytical solution (Eq. 10) sampled at time intervals of 0.01 until time $t=1.0$, with a validation period from $1.0\le t \le 1.25$. In all the experiments, we use both ${\mathcal {L}}_1$ and ${\mathcal {L}}_2$ regularization for the weights of the neural network, and prune them if their value drops below a certain threshold (only if the weightage of ${\mathcal {L}}_1$ regularization is non-zero), in order to promote sparsity. The set of tuned hyperparameters used to generate the results presented next are provided in the supplementary information, Sect. SI-2.2. Given the analytical solution data, $\{u^{true}(x, T_i), \; -L \le x \le L \}_{i=1}^M$, the loss function is based on time and space averaged mean-absolute-error (MAE), ${\mathcal {L}} = \frac{1}{M} \sum _{i=1}^M \int _{-L}^L \frac{1}{2L} |u^{pred}(x, T_i) - u^{true}(x, T_i)| dx$, where $M=100$ is the number of high-fidelity solution states at different times available for training.

*Learning results* We perform six repeats of the experiment with exactly the same set of hyperparameters, and the learned model with the mean and standard deviation of the weights is as follows,

13 $$\begin{aligned} \begin{aligned} \frac{\partial u}{\partial t} = -u\frac{\partial u}{\partial x} - (4.9680 \pm 0.0008) u\frac{\partial u}{\partial x} - (1.0105 \pm 0.0002)\frac{\partial ^3 u}{\partial x^3}. \end{aligned} \end{aligned}$$

The true coefficients corresponding to the learned $u\frac{\partial u}{\partial x}$ and $\frac{\partial ^3 u}{\partial x^3}$ terms are $-5.0$ and $-1.0$, respectively. The learned closure is able to recover the true model, and the slight discrepancy in the learned coefficients is to compensate for the very small discretization and truncation errors. To illustrate this, we compare the root-mean-square-error (RMSE), ${\mathcal {L}} = \frac{1}{M} \sum _{i=1}^M \sqrt{ \sum _{j=1}^{N_x} \frac{1}{N_x} (u^{pred}(x_j, T_i) - u^{true}(x_j, T_i))^2}$, of the learned closure and the true model solved using the same numerical schemes. The RMSE (mean and standard deviation) obtained for the learned closure and the true model solved numerically is $0.0063 \pm 0.0014$ and 0.0251, respectively. Thus, on average, the learned closure leads to a smaller RMSE than the error of the numerically-solved true model. We note that this excellent accuracy in the coefficients of the recovered (learned) model compared to the true model is similar to that observed in SINDy and its variants for the KdV PDE in^32–34^.

*Sensitivity* The learning was sensitive to batch-time, and higher values were especially detrimental to convergence. This behavior is in general observed when the error between the low- and high-fidelity models is large, e.g., when there is no low-fidelity model. Using a smaller batch size and regularization weights lead to slightly different values of the learned coefficients. This is especially noted for the $u^2\frac{\partial u}{\partial x}$ term, whose weight tends towards a non-zero value with a very small magnitude. For a study on the impact of different hyperparameters (encountered specifically in the nCM framework and SciML in general) on training, we refer to^55^. In the current set of experiments, the learning framework is able to recover the known true model and, due to this, we do not additionally focus on demonstrating generalization over initial conditions, boundary conditions, and grid resolution.

### Experiments 1b: advecting shock—model discovery and generalization

In the second set of experiments, we employ the advecting shock PDE models. First, a *g*nCM discovers the leading truncation term and a correction to the nonlinear advection term by interpreting the learned Markovian neural closure. Second, we utilize both Markovian and non-Markovian *g*nCM terms trained on data corresponding to just a few combinations of grid resolution and Reynolds number, and demonstrate the generalization of the learned closure model over grid resolution, Reynolds number, initial and boundary conditions, along with superior performance compared to the popular Smagorinsky closure model. We further interpret the learned closure by analysing the weights of the learned neural networks, and find the closure to be independent of the Reynolds number despite it being one of the functional inputs.

*Setup: true model, data generation, and low-fidelity model* We consider the classic form of the Burgers equation as the governing high-fidelity model,

14 $$\begin{aligned} \begin{aligned} \frac{\partial u}{\partial t} = -u\frac{\partial u}{\partial x} + \nu \frac{\partial ^2 u}{\partial x^2} , \quad 0 \le x \le L, ~ t \in (0, T] , \end{aligned} \end{aligned}$$

where $\nu$ is the diffusion coefficient. The data is generated from an analytical solution of this Burgers equation  (14) with initial and boundary conditions,

15 $$\begin{aligned} \begin{aligned} u(x, 0)&= \frac{x}{1 + \sqrt{\frac{1}{t_0}} \exp \left( Re\frac{x^2}{4} \right) }, \quad u(0, t) = 0, \quad \text {and} \quad \frac{\partial u(x, t)}{\partial x}\bigg |_{x=L} = 0 , \end{aligned} \end{aligned}$$

where the Reynolds number $Re = 1/\nu$ and $t_0 = \exp (Re/8)$. This solution is given by,

16 $$\begin{aligned} \begin{aligned} u(x, t)&= \frac{x / (t+1)}{1 + \sqrt{\frac{t+1}{t_0}} \exp \left( Re\frac{x^2}{4t + 4} \right) } . \end{aligned} \end{aligned}$$

However, when the discrete version of the above Eq. (14) is solved numerically, truncation and round-off errors occur and the numerical solution incurs discretization errors^35,56^.

**Figure 3:**
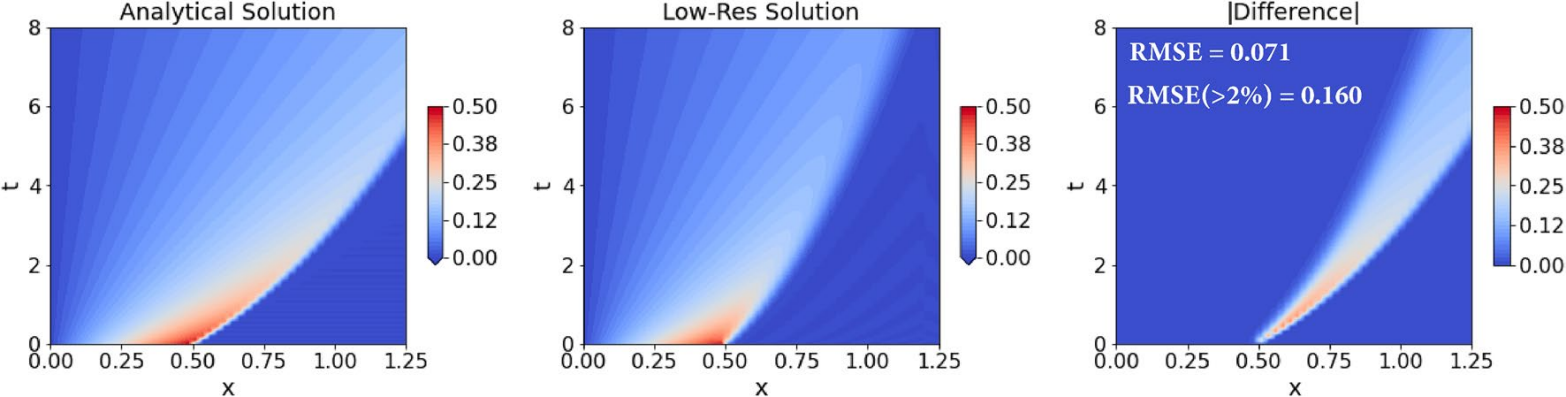
Comparison of the numerical solution of the Burgers equation (with $Re=1000$) on a low-resolution grid (Eqs. 14 and 15; low-fidelity model; middle plot), with its corresponding analytical solution (Eq. 16; high-fidelity model; left plot). The low-fidelity model is solved on a grid with $N_x=50$ grid points, and the absolute difference between the two solutions is provided in the right plot. We also provide a pair of time-averaged errors, specifically: root-mean-squared-error (RMSE); and RMSE considering only the grid points where the error is at least $2\%$ of the maximum velocity value, denoted by RMSE($>2\%$).

*Numerics* We solve the Burgers equation (14) numerically with the following schemes: 1st order accurate upwind for the advection term, 2nd order accurate central-difference for the diffusion term, and *Vode* scheme for adaptive time-stepping. Thus, the leading order truncation error term is given by, $-\frac{\Delta x}{2} u \frac{\partial ^2 u}{\partial x^2} + {\mathcal {O}}(\Delta x^2)$, where $\Delta x$ is the uniform grid-spacing. The terms in ${\mathcal {O}}(\Delta x^2)$ contain spatial derivatives of order 3 and above. *Comparison LF-HF:* A comparison of the analytical (Eq. 16) and numerical solution of the Burgers equation is provided in Fig. 3. One can clearly notice the effects of numerical diffusion and the error in the location of the shock peak at later times due to truncation errors.

#### Learning interpretable truncation errors and nonlinear flux corrections

*Training: NN architecture, data, and loss function* First, we only consider a Markovian closure term based on a library composed of second-degree combinations of *u*, $\frac{\partial u}{\partial x}$, and $\frac{\partial ^2 u}{\partial x^2}$. The library explicitly omits $u\frac{\partial u}{\partial x}$ because it is already known as part of the governing equation, and $u^2$ because it cannot be part of truncation error due to the absence of any derivative. Hence, the Markovian closure term is assumed to be a linear combination of $\left\{ \right. \Delta x \left( \frac{\partial u}{\partial x} \right) ^2, \Delta x^3 \left( \frac{\partial ^2 u}{\partial x^2} \right) ^2, \Delta x^2 \left( \frac{\partial u}{\partial x} \frac{\partial ^2 u}{\partial x^2} \right) , \Delta x \left( u \frac{\partial ^2 u}{\partial x^2} \right) \left. \right\}$, of which the fourth term is true but unknown leading order truncation error term itself, and other terms are informed but still expected to be redundant. Each of the terms is multiplied with appropriate powers of $\Delta x$, such that the closure terms are dimensionally consistent with the other terms of the Burgers equation. The 4th order accurate central and upwind finite-difference schemes^53^ are used to compute the spatial derivatives in the Markovian closure, so as to eliminate additional sources of truncation error from our analysis. The training data consists of the analytical solution up until $T=4.0$ solved in a domain of length $L=1.25$ and saved at every 0.01 time-intervals, for three different combinations of $N_x$ (number of grid points in $x-$direction) and *Re*. The chosen ($N_x$, *Re*) pairs, $\{(100, 50), (150, 750), ~\text {and} ~ (200, 1250) \}$, are such that the $-\frac{\Delta x}{2} u \frac{\partial ^2 u}{\partial x^2}$ term is really the leading source of error. In every epoch, we parse through the training data of each of these pairs, selected in random order by sampling without replacement. We tune the hyperparameters based on performance in the training period ($0.0 \le t \le 4.0$) and the validation period ($4.0 \le t \le 6.0$), and these are provided in Sect. SI-2.2. The Markovian closure model is a simple neural network with no hidden layers and only linear activation in the output layer, in-effect equivalent to a linear combination of the inputs. Given the analytical solution, $\{u^{true}(x, T_i), \; 0 \le x \le L \}_{i=1}^M$, the loss function is once again the time and space averaged mean-absolute-error (MAE), ${\mathcal {L}}= \frac{1}{M} \sum _{i=1}^M \int _{0}^L \frac{1}{L} |u^{pred}(x, T_i) - u^{true}(x, T_i)| dx$, where $M=400$ is the number of high-fidelity solution states at different times available for training.

*Learning results* We perform eight repeats of the same experiment with the tuned hyperparameters. The resulting learned model with the mean and standard deviation of the coefficients is as follows,

17 $$\begin{aligned} {\mathcal {F}}_{NN} \left( \Delta x \left( \frac{\partial u}{\partial x} \right) ^2, \Delta x^3 \left( \frac{\partial ^2 u}{\partial x^2} \right) ^2, \Delta x^2 \left( \frac{\partial u}{\partial x} \frac{\partial ^2 u}{\partial x^2} \right) , \Delta x \left( u \frac{\partial ^2 u}{\partial x^2} \right) ; \phi \right) \\&\quad = (0.133 \pm 0.017) \Delta x \left( \frac{\partial u}{\partial x} \right) ^2 + (0.009 \pm 0.023)\Delta x^3 \left( \frac{\partial ^2 u}{\partial x^2} \right) ^2 \\&\quad - (0.323 \pm 0.022) \Delta x \left( u \frac{\partial ^2 u}{\partial x^2} \right) . \end{aligned}$$

**Figure 4:**
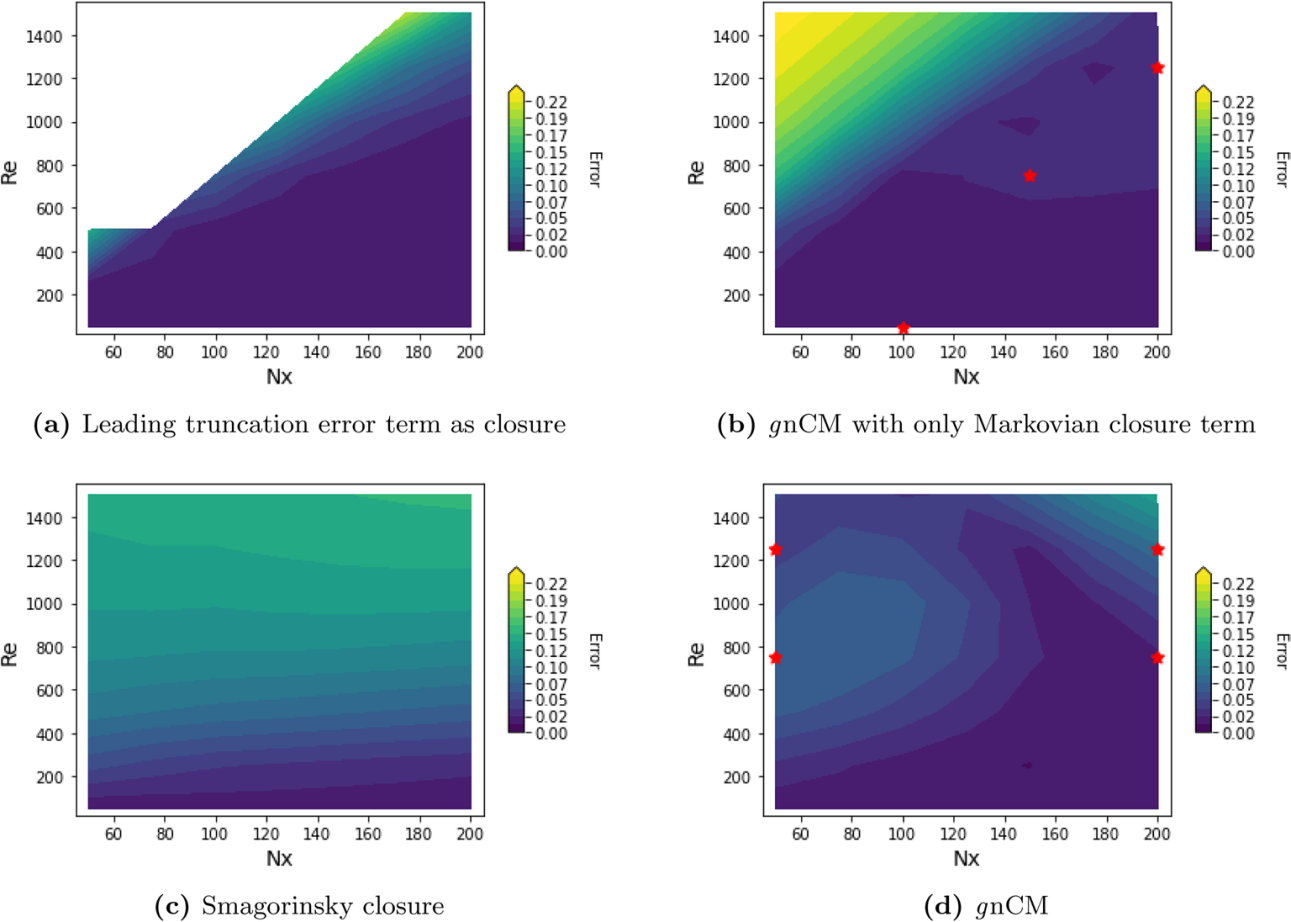
Performance of four closure models for the Burgers equation (Eqs. 14 and 15) evaluated for various $(N_x, Re)$ pairs in the 2D domain spanned by $50 \le N_x \le 200$ and $50 \le Re \le 1500$. The error provided is the $RMSE\,(>2\%)$ (see Fig. 3 for description) computed w.r.t. the corresponding analytical solutions (Eq. 16) for $0.0\le t \le 8.0$ in a domain of length $L = 1.25$. (**a)** Leading truncation error term, $-\frac{\Delta x}{2} u \frac{\partial ^2 u}{\partial x^2}$, as closure. The white region in the top-left denotes an unconverged numerical solution; (**b)** Learned *g*nCM with only the Markovian term, with the three ’s marking the ($N_x, Re$) pairs used as training data; (**c**) Smagorinsky LES model with $C_s = 1.0$; (**d**) Learned *g*nCM with both Markovian and non-Markovian closure terms, with the four ’s marking the ($N_x, Re$) pairs used as training data.

For evaluation, we first compare the performance of this learned *g*nCM w.r.t. using the true leading truncation error term ($-\frac{\Delta x}{2} u \frac{\partial ^2 u}{\partial x^2}$) as the closure itself. For both cases, we evolve the Burgers equation with the respective closure terms up until $T=8.0$ (beyond training and validation time-periods), for 35 $(N_x, Re)$ pairs in the 2D domain spanned by $50 \le N_x \le 200$ and $50 \le Re \le 1500$. In Fig. 4 we provide the RMSE$(>2\%)$ error (see Fig. 3 for description). When the true leading truncation error term is used as the closure, we find that increasing *Re* and lowering $N_x$ values leads to instabilities in the solution which causes it to explode. On the contrary, in the learned *g*nCM case, even though it was not shown any training data in the high *Re* and low $N_x$ regime, it still provides a stable solution, and, on average, performs better than its counterpart in the other regions of the $(N_x, Re)$ domain. To interpret the learned closure further, we rewrite it by substituting, $\frac{\partial }{\partial x}\left( u\frac{\partial u}{\partial x} \right) = \left( \frac{\partial u}{\partial x} \right) ^2 + \left( u \frac{\partial ^2 u}{\partial x^2} \right)$ in Eq. (17),

18 $$\begin{aligned}{\mathcal {F}}_{NN} \left( \Delta x \left( \frac{\partial u}{\partial x} \right) ^2, \Delta x^3 \left( \frac{\partial ^2 u}{\partial x^2} \right) ^2, \Delta x^2 \left( \frac{\partial u}{\partial x} \frac{\partial ^2 u}{\partial x^2} \right) , \Delta x \left( u \frac{\partial ^2 u}{\partial x^2} \right) ; \phi \right) \\&\quad = (0.133 \pm 0.017) \Delta x \frac{\partial }{\partial x}\left( u\frac{\partial u}{\partial x} \right) + (0.009 \pm 0.023)\Delta x^3 \left( \frac{\partial ^2 u}{\partial x^2} \right) ^2 \\&\quad - (0.456 \pm 0.012) \Delta x \left( u \frac{\partial ^2 u}{\partial x^2} \right) . \end{aligned}$$

Thus, the learned *g*nCM contains the $\Delta x \left( u \frac{\partial ^2 u}{\partial x^2} \right)$ term with a coefficient of correct sign but slightly smaller value—in absolute value—in comparison to that of the true leading truncation error term. Along with that, the other significant term, $\Delta x \frac{\partial }{\partial x}\left( u\frac{\partial u}{\partial x} \right)$, corresponds to a first-order Taylor series correction to the nonlinear advection term, and can help with mitigating the resolution error highlighted earlier. Finally, it is remarkable that the important $\Delta x \frac{\partial }{\partial x}\left( u\frac{\partial u}{\partial x} \right)$ term was missing from the input features; however, to our surprise, it is still accounted for indirectly in the learned closure, utilizing the redundant terms present in the input feature library. This highlights a noteworthy learning capability of the *g*nCM.

#### Learning generalizable and interpretable closures

*Training: NN architecture, data, and loss function* Keeping the Markovian closure term formulation of Sect. SI-2.2, we now add the non-Markovian closure term with inputs, $\{u, \frac{\partial u}{\partial x}, \frac{\partial ^2 u}{\partial x^2}, \nu , \Delta x\}$, discretized using $4^{th}$ order finite-difference schemes, and the deep-NN architecture given in Table SI-1. We utilize a fully-connected deep-NN with four hidden-layers and the non-linear *swish* activation. The output of the NN is multiplied with |*u*| to ensure that the contribution of the non-Markovian closure term is zero in the right-hand parts of the domain where the shock is yet to reach. As the non-Markovian closure term is nonlinear, we do not explicitly make the inputs dimensionally consistent with other terms in the Burgers equation. The overall training and evaluation setup are as in “Learning interpretable truncation errors and nonlinear flux corrections”, however, this time four pairs of $(N_x, Re)$ are used such that all four combinations of high and low $N_x$ and *Re* are contained in the training data. The chosen pairs were, $\{(50, 750), (200, 750), (50, 1250), (200, 1250)\}$. The tuned set of hyperparameters is provided in Sect. SI-2.2. The time-delay, $\tau = 0.075$, is based on the optimal-time delay established for the Burgers equation experiments in^2^.

*Learning results* We perform seven repeats of the experiment with exactly the same set of tuned hyperparameters. The learned coefficients for the Markovian term are different than those in Eq. (17) due to the presence of the non-Markovian term, however, once again, the most weightage is given to the $\Delta x \left( \frac{\partial u}{\partial x} \right) ^2$ and $\Delta x \left( u \frac{\partial ^2 u}{\partial x^2} \right)$ terms. Upon inspection, the weights of the input layer of the deep-NN in the non-Markovian term being multiplied with $\nu$ were consistently found to be particularly small (${\mathcal {O}}(10^{-4})$), indicating that the learned closure is independent of $\nu$. For one of the experiment runs, we show in Fig. 4 the performance for $(N_x, Re)$ pairs in the 2D domain spanned by $50 \le N_x \le 200$ and $50 \le Re \le 1500$, and compare it with that of the popular Smagorinsky model used for subgrid-scale turbulence closure in large eddy simulations (LES). To the Burgers equation (14), this model introduces a dynamic turbulent eddy viscosity ($\nu _e$) resulting in,

19 $$\begin{aligned} \begin{aligned} \frac{\partial u}{\partial t} = -u\frac{\partial u}{\partial x} + \nu \frac{\partial ^2 u}{\partial x^2} + \frac{\partial }{\partial x}\left( \nu _e\frac{\partial u}{\partial x}\right) , \end{aligned} \end{aligned}$$

where $\nu _e = (C_s \Delta x)^2 \big | \frac{\partial u}{\partial x}\big |$ and $C_s$ is the Smagorinsky constant. As the rectangle formed by the training $(N_x, Re)$ pairs is only a subset of the rectangle in which we evaluate the learned closure, we are testing both interpolation and extrapolation performance w.r.t. changing the physical parameter governing the model and grid resolution. We find that the learned *g*nCM clearly outperforms the Smagorinsky model. It should be noted, that in Fig. 4d, the bottom-right corner (low *Re* and high $N_x$ region) has inherently small errors even without the presence of a closure. Further, the amount of error between low-fidelity and high-fidelity solutions is different for the four training data $(N_x, Re)$ combinations; for example, (50, 1250) (coarsest resolution, higher Re) should incur the most error. Thus, we notice a differential in the impact of learned *g*nCM in reducing the error at and around different training data $(N_x, Re)$ pairs.

**Figure 5:**
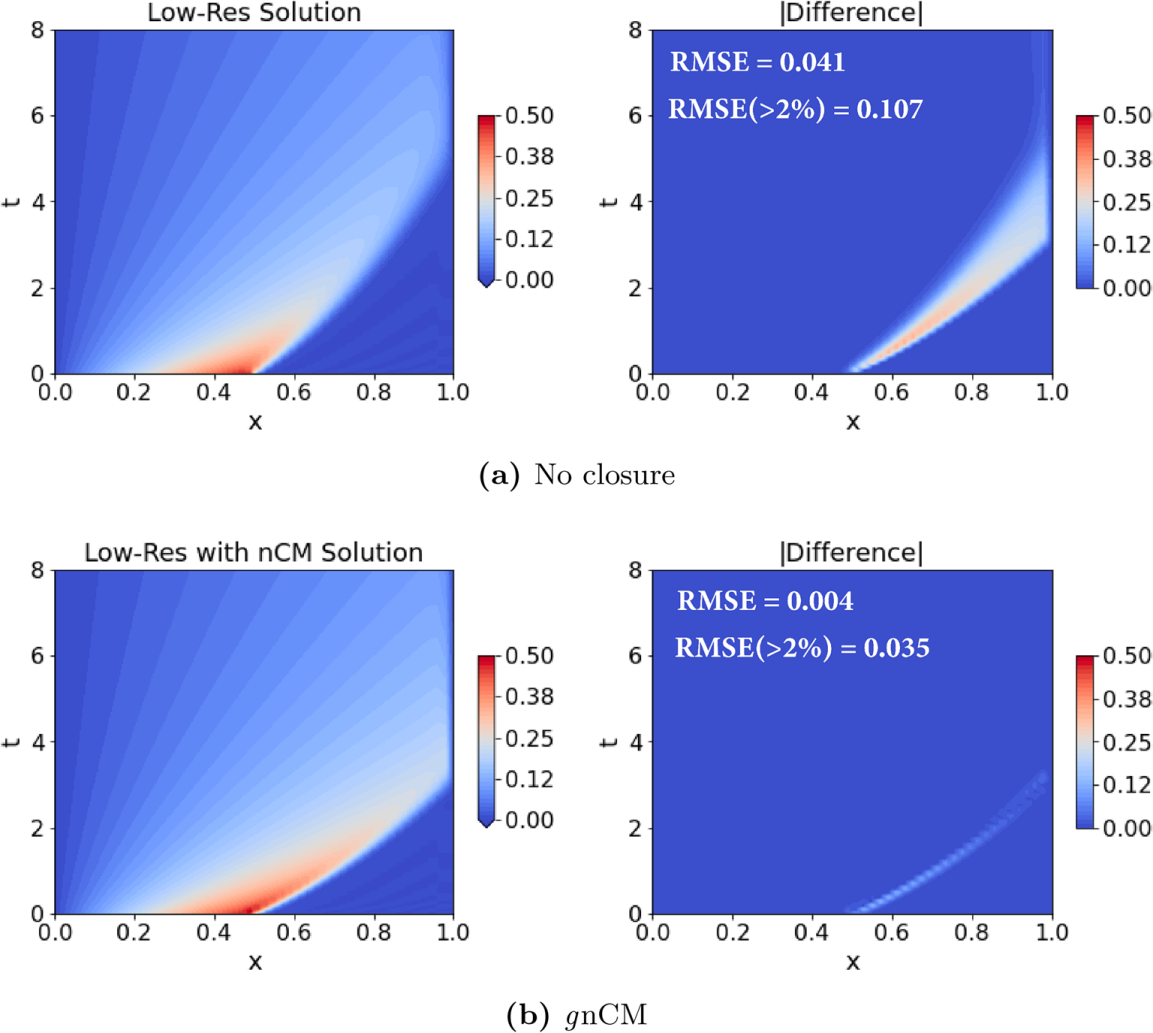
Solution of the Burgers equation with and without the learned generalized neural closure model (*g*nCM) for $Re = 1000$, a low-resolution grid ($N_x = 50$), and zero Dirichlet boundary condition on the right edge. For each case, we also provide the pair of time-averaged errors (see Fig. 3 for description).

As claimed earlier, we expect the learned *g*nCM to be also generalizable over different boundary conditions. We tested this by modifying the boundary conditions. The analytical solution (Eq. 16) used in training corresponded to Neumann boundary conditions on the right edge of the domain. This was changed to a zero Dirichlet boundary condition. Furthermore, the length of the domain was decreased to $L=1$, and $N_x = 50$ number of equally-spaced grid points were used in our low-fidelity model with $Re=1000$. Since no closed-form analytical solution exists for the Dirichlet boundary conditions case, we solve the system with $N_x = 1000$ grid points and use that as the true solution for comparing the performance of our learned closure. In Fig. 5, we find that the learned *g*nCM is able to keep the errors remarkably low throughout the time period encompassing training, testing, and prediction.

*Sensitivity* In general, the quality of learning was less sensitive to the batch-time hyperparameter, however, higher values led to more interpretable closures. Using lower-order finite-difference schemes for the closure inputs did not compromise the performance of the learned closures, however, it did lead to a decrease in interpretability. Sensitivity to other hyperparameters was similar to that observed in Experiments-1a.

### Experiments 2a: ocean acidification—interpretable model discrimination

In the third set of experiments, we consider coupled physical–biological–carbonate PDE models that are used to study ocean acidification (OA). We utilize the Markovian neural closure model to interpretably discriminate between candidate functional forms of uncertain Zooplankton mortality term.

*Setup: True model, data generation, and low-fidelity model* OA models are used to study and predict essential carbonate chemistry and biological production cycles, and their interplay with global warming. A plethora of biogeochemical models have been proposed. They differ in their complexity, or ability to resolve different biological processes. A set of parameter values and functional forms that might work in a particular ocean region may not apply anywhere else. As an additional source of complexity, there may be seasonal variability in these functional forms^57–59^.

For this set of experiments, the high-fidelity model is similar to the Hadley Centre Ocean Carbon Cycle (HadOCC) model^60^, where the biological part is a modified version of four components [nutrients (N), phytoplankton (P), zooplankton (Z), and detritus (D)] developed in^61^ for the Gulf of Maine, along with dissolved inorganic carbon (DIC) and total alkalinity (TA) for the carbonate part. The NPZD model is,

20 $$\begin{aligned} \begin{aligned} \frac{dN}{dt}&= -U_P + \lambda G_Z + \varepsilon D , \\ \frac{dP}{dt}&= U_P - G_Z - m_P P , \\ \frac{dZ}{dt}&= \gamma G_z - M_Z(Z) , \\ \frac{dD}{dt}&= (1-\gamma -\lambda ) G_Z + m_P P + M_Z(Z) - \varepsilon D , \end{aligned} \end{aligned}$$

where $U_P$ is the phytoplankton growth, regulated by nitrogen limitation based on Michaelis–Menten kinetics (*f*(*N*)) and photosynthetically active radiation (*f*(*I*)), and $G_Z$ the zooplankton grazing, each given by,

21 $$\begin{aligned} \begin{aligned} U_P&= \mu _{max} f(N) f(I) P, \quad f(N) = \frac{N}{N+K_N}, \\ f(I)&= (1-\exp (\alpha I / \mu _{max})) \exp (-\beta I / \mu _{max}) \\ I(z)&= I_0 \exp (-k_W z), \quad G_Z = \frac{g_{max} ZP^2}{P^2 + K_P^2}, \end{aligned} \end{aligned}$$

and $M_Z(Z)$ is the to-be-learned zooplankton mortality. In these equations, the concentration of biological variables is measured in nitrogen (mmol N m$^{-3}$), *z* is depth, and the other parameters are: $\mu _{max}$, maximum growth rate of phytoplankton; $K_N$, half-saturation constant; $\alpha$ and $\beta$, light-growth slope and inhibition coefficient; $I_0$, photosynthetically active radiation (PAR) at the sea surface; $k_W$, attenuation coefficient of water; $g_{max}$, zooplankton maximum grazing rate; $K_P$, half-saturation constant for zooplankton grazing; $\gamma$, assimilation coefficient; $m_z$, zooplankton mortality coefficient; $m_p$, phytoplankton mortality coefficient; $\lambda$, active respiration zooplankton expressed as a fraction of grazing; and $\varepsilon$, remineralization rate of detritus. The carbon in the system is coupled with the nitrogen by fixed carbon–nitrogen ratios, $C_P$, $C_Z$, and $C_D$,

22 $$\begin{aligned} \begin{aligned} \frac{d (DIC)}{dt}&= -C_P \frac{dP}{dt} - C_Z \frac{dZ}{dt} - C_D \frac{dD}{dt} - \gamma _c C_P U_P , \\ \frac{d(TA)}{dt}&= -\frac{1}{\rho _w}\frac{dN}{dt} - \frac{2\gamma _c C_P U_P}{\rho _w} , \end{aligned} \end{aligned}$$

and neither DIC nor TA has any effect on the biology because phytoplankton growth is not carbon limited. The last term in the DIC equation represents the precipitation of calcium carbonate to form shells and other hard body parts, which subsequently sink below the euphotic zone, also known as “hard flux”. This flux is modeled to be proportional (and additional) to the uptake of carbon for primary production. The chemistry dictates the decrease in total alkalinity by two molar equivalents for each mole of carbonate precipitated. In general, since TA is measured in mmol  C  kg$^{-1}$ (or $\mu$ mol  C  kg$^{-1}$), we divide the right-hand-side (RHS) of the TA equation by the density of sea-water ($\rho _w$). Moreover, the units of DIC concentration are mmol  C m$^{-3}$.

**Figure 6:**
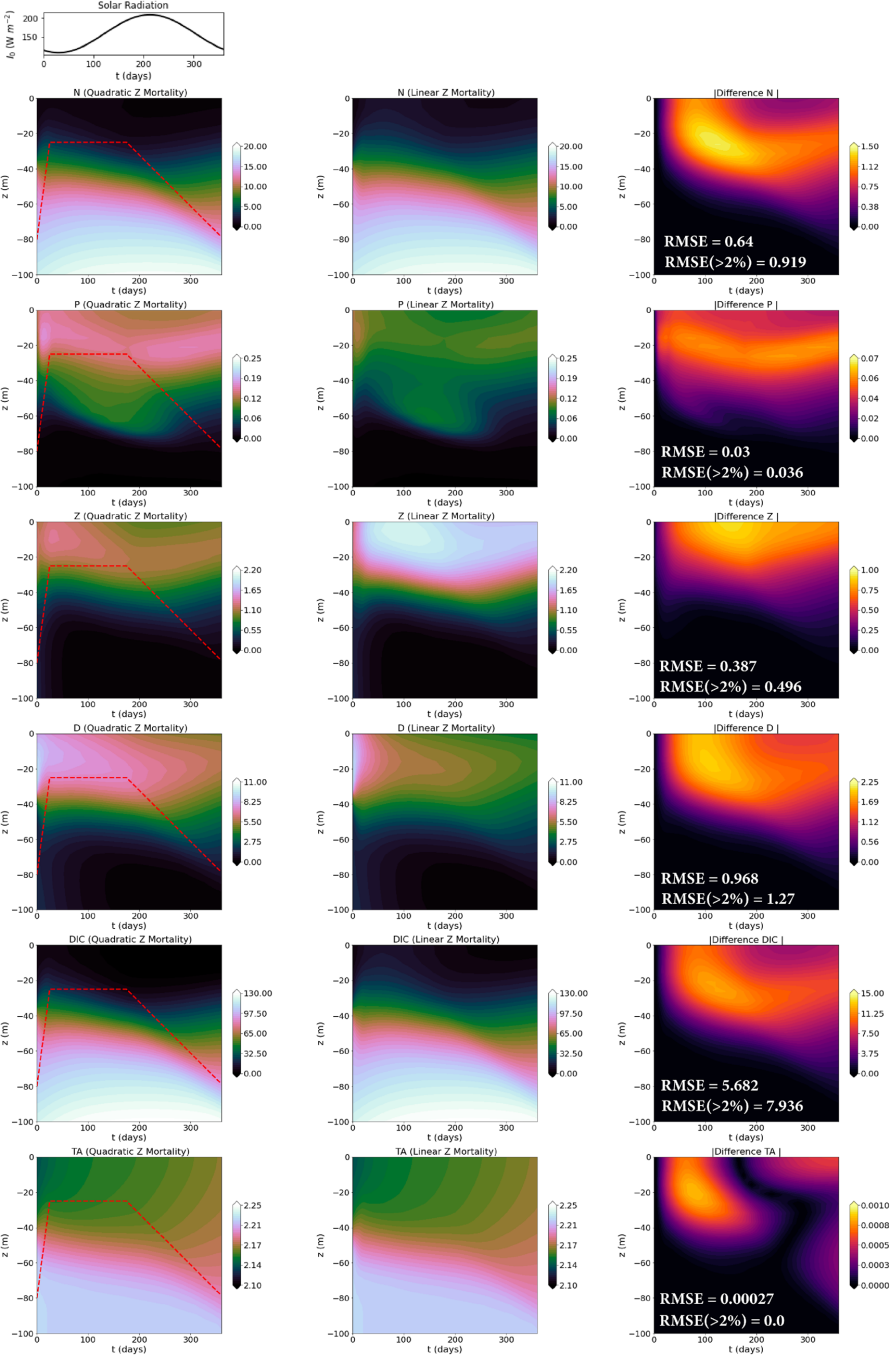
Solutions (in each column, concentration profiles of *N*, *P*, *Z*, *D* in mmol N m$^{-3}$, *DIC* in mmol C m$^{-3}$, and *TA* in mmol C kg$^{-1}$, all vs. time in days) of the OA model used in Experiments-2a, corresponding to different functional forms for the zooplankton mortality term. Left-column: the top panel shows the yearly variation of solar radiation and the subsequent panels depict the states from the NPZD-OA model with $M_Z(Z) = \frac{m_Z}{2}(Z + Z^2)$ (ground truth), overlaid with the dynamic mixed layer depth in dashed red lines; middle-column: states from the NPZD-OA model with $M_Z(Z) = \frac{m_Z}{2}Z$ (low-fidelity); right-column: absolute difference between the corresponding states in the left- and middle- column. For each case, we also provide the pair of time-averaged errors (see Fig. 3 for description).

The above biological and carbonate models are often coupled with physical models to introduce both spatial and temporal components. For our experiments, we use a 1-D diffusion-reaction PDE with vertical eddy mixing parameterized by the operator $\partial /\partial z \left( K_z(z, M)\partial /\partial z (\bullet ) \right)$, where $K_z$ is a dynamic eddy diffusion coefficient. A mixed layer of varying depth ($M = M(t)$) is used as a physical input to the OA models. Thus, each biological and carbonate state variable *B*(*z*, *t*) is governed by the following non-autonomous PDE,

23 $$\begin{aligned} \frac{\partial B}{\partial t}= & {} S^B + \frac{\partial }{\partial z}\left( K_z(z, M(t))\frac{\partial B}{\partial z}\right) , \end{aligned}$$

24 $$\begin{aligned} K_z(z, M(t))= & {} K_{z_b} + \frac{(K_{z_0} - K_{z_b})(\arctan (-\gamma _t (M(t) - z)) - \arctan (-\gamma _t (M(t) - D_z)))}{\arctan (-\gamma _t M(t)) - \arctan (-\gamma _t (M(t) - D_z))} \;, \end{aligned}$$

where $K_{z_b}$ and $K_{z_0}$ are the diffusion at the bottom and surface, respectively, $\gamma _t$ is the thermocline sharpness, and $D_z$ is the total depth. The 1-D model and parameterizations are adapted from^62^ and^63^. They simulate the seasonal variability in upwelling, sunlight, and biomass vertical profiles. The dynamic mixed layer depth, surface photosynthetically-available radiation $I_0(t)$, and biomass fields *B*(*z*, *t*) are shown in Fig. 6. The radiation $I_0(t)$ and total biomass concentration, $T_{bio}(z,t)$, affects $S^B$ and the initial conditions.

To generate data, we first initialize the *N* state with the depth-varying total biomass concentration and the *P*, *Z*, and *D* states with zero concentrations, and then run a one-month spin-off of just the NPZD model without the diffusion term and a constant sea-surface solar radiation in order to determine the stable equilibrium of the biological states. These equilibrium states form the initial conditions for the respective states in the NPZD-OA model. To initialize *DIC*, we multiply the equilibrium state for *N* with the nitrogen-to-carbon ratio that is considered nearly equal to the value of $C_P$. *TA* is often assumed to have a dependence on salinity and biological processes^64^. The contribution from salinity (*S* in *PSU*) is modeled using a linear relationship optimized for the Gulf of Maine, $$TA = {\left\{ \begin{array}{ll} (198.10 + 61.75 S)/1000 \;, &{} S < 32.34 \\ (744.41 + 44.86 S)/1000 \;, &{} S \ge 32.34 \end{array}\right. }$$ (Dr. P.J. Haley Jr., *pers. comm.*), while the biological impact is given by Eq. (22). We assume a stationary salinity profile described using a sigmoid function $S(z) = A + \frac{K - A}{(C + Q \exp (-Bz))^{1/\nu }}$ with $A = 31.4~PSU$, $K = 32.8~PSU$, $C = 1.0$, $Q = 0.5$, $B = 0.25$, and $\nu = 2.0$. Thus, we can initialize TA based on salinity and evolve it using Eq. (22) coupled with Eqs. (23, 24).

For the low-fidelity model, we assume that we have only prior knowledge about the existence of a linear zooplankton mortality term, i.e., $M_Z(Z) = \frac{m_Z}{2} Z$. For the high-fidelity model, however, the true zooplankton mortality contains an additional quadratic dependence, i.e., $M_Z(Z) = \frac{m_Z}{2} (Z + Z^2)$.

*Numerics* We use a 2nd order central difference scheme for the spatial discretization ($N_z = 20$), and *dopri5*^65^ scheme for time integration with adaptive time-stepping. *Comparison LF-HF:* in Fig. 6-left- and -mid-columns, we provide a year-long simulation for the NPZD-OA model with quadratic (truth) and linear (prior) *Z* mortality terms, respectively. We notice the low *Z* concentration and enhanced *P* bloom in the former case. Figure 6-right-column provides the absolute difference between the two cases. Values of the model parameters are provided in Sect. SI-2.

*Training: NN architecture, data, and loss function* For the *g*nCM—we only consider the Markovian term—belonging to a linear combination of a library of popular mortality functions^66^, $\{Z, Z^2, \frac{Z^2}{1+Z}, \exp {Z}\}$. Compared to the true zooplankton mortality term, our library contains three superfluous or redundant terms, *Z*, $\frac{Z^2}{1+Z}$, and $\exp {Z}$, noting that the *Z* term is already a part of the low-fidelity model and completely known. For the Markovian term, we use again a simple NN with no hidden layers and linear activation in the output layer. Using weight constraints for the output layer, we enforce biomass conservation in the *N*, *P*, *Z*, and *D* equations and couple with *DIC* and *TA* equations as in the known system (Eqs. 20 and 22 ). Architectural details are given in Table SI-1 and the tuned set of training hyperparameters in Sect. SI-2.2. The training data consists of the true/high-fidelity model solution sampled at time intervals of 0.1 day, until $t=$30 days, $\{\, \{B^{true}(z, T_i)\}_{B\in \{N, P, Z, D, DIC, TA\}}\}_{i=1}^M$, i.e., a $M=300$ high-fidelity solution states. We use an MAE-based loss function, ${\mathcal {L}} = \frac{1}{M} \sum _{i=1}^M \int _0^{D} \frac{1}{D} \sqrt{\sum _{B \in \{N, P, Z, D, DIC, TA\}} \frac{1}{\sigma _{B}}|B^{pred}(z, T_i) - B^{true}(z, T_i)|} dz$. Here, $\sigma _B$’s are hyperparameters to scale the importance of different state variables based on their magnitudes. After multiple hyperparameter tuning experiments, values of $\sigma _N = 1, ~\sigma _{P} = 0.25, ~\sigma _Z = 1, ~\sigma _D = 1, ~\sigma _{DIC} = 2, ~\sigma _{TA} = 0.1$, were found to aid in learning.

*Learning results* In seven repeats of the experiment with exactly the same hyperparameters, the learned models consisted of no contribution of the closure to the *N*, *P*, and *TA* equations, while for the *Z*, *D*, and *DIC* equations the contributions were found—with mean and standard deviation—to be $(-0.02996 \pm 0.00014) Z^2$, $(0.03001 \pm 0.00013) Z^2$, and $(-0.05603 \pm 0.00136) Z^2$, respectively. For reference, the true contribution of the zooplankton quadratic mortality term to the *Z*, *D*, and *DIC* equations are given as $-0.02998 Z^2$, $0.02998 Z^2$, and $-0.05621 Z^2$, respectively.

*Sensitivity* Multiple experiments were done to study the effects of hyperparameters, such as batch-time, batch-size, regularization factors, etc., and the convergence to the true model was the most severely compromised when increasing batch-time and changing the loss-scaling for individual state variables.

### Experiments 2b: ocean acidification—model complexity

In the last set of experiments, we again consider the coupled physical–biological–carbonate PDE models, however, this time we utilize the full generalized neural closure model to augment a simpler model obtained by aggregation of components and other simplifications of processes and parameterizations, such that, it becomes as accurate as the more complex model. Simultaneously, we also discriminate between the candidate functional forms of the uncertain Zooplankton mortality term in an interpretable fashion.

*Setup: true model, data generation, and low-fidelity model* The high-fidelity model and the data are those used in Experiments-2a, where we model the intermediate state of detritus, thus capturing processes such as remineralization and quadratic zooplankton mortality, i.e., $M_Z(Z) = \frac{m_Z}{2}(Z + Z^2)$. The low-fidelity model is the less complex three-component NPZ model,

25 $$\begin{aligned} \begin{aligned} \frac{dN}{dt}&= -U_P + (1-\gamma ) G_Z + m_P P + \frac{m_Z}{2} Z , \\ \frac{dP}{dt}&= U_P - G_Z - m_P P , \\ \frac{dZ}{dt}&= \gamma G_z - \frac{m_Z}{2} Z , \end{aligned} \end{aligned}$$

coupled with the carbonate system using fixed carbon-nitrogen ratios, $C_P$, and $C_Z$,

26 $$\begin{aligned} \begin{aligned} \frac{d (DIC)}{dt}&= -C_P \frac{dP}{dt} - C_Z \frac{dZ}{dt} - \gamma _c C_P U_P , \\ \frac{d(TA)}{dt}&= -\frac{1}{\rho _w}\frac{dN}{dt} - \frac{2\gamma _c C_P U_P}{\rho _w} , \end{aligned} \end{aligned}$$

and with the 1-D diffusion-reaction PDE (23). The goal of these experiments is to use the *g*nCM to simultaneously learn the functional form of the zooplankton mortality term using the Markovian closure term, and account for the missing intermediate state of detritus through the non-Markovian closure term.

**Figure 7:**
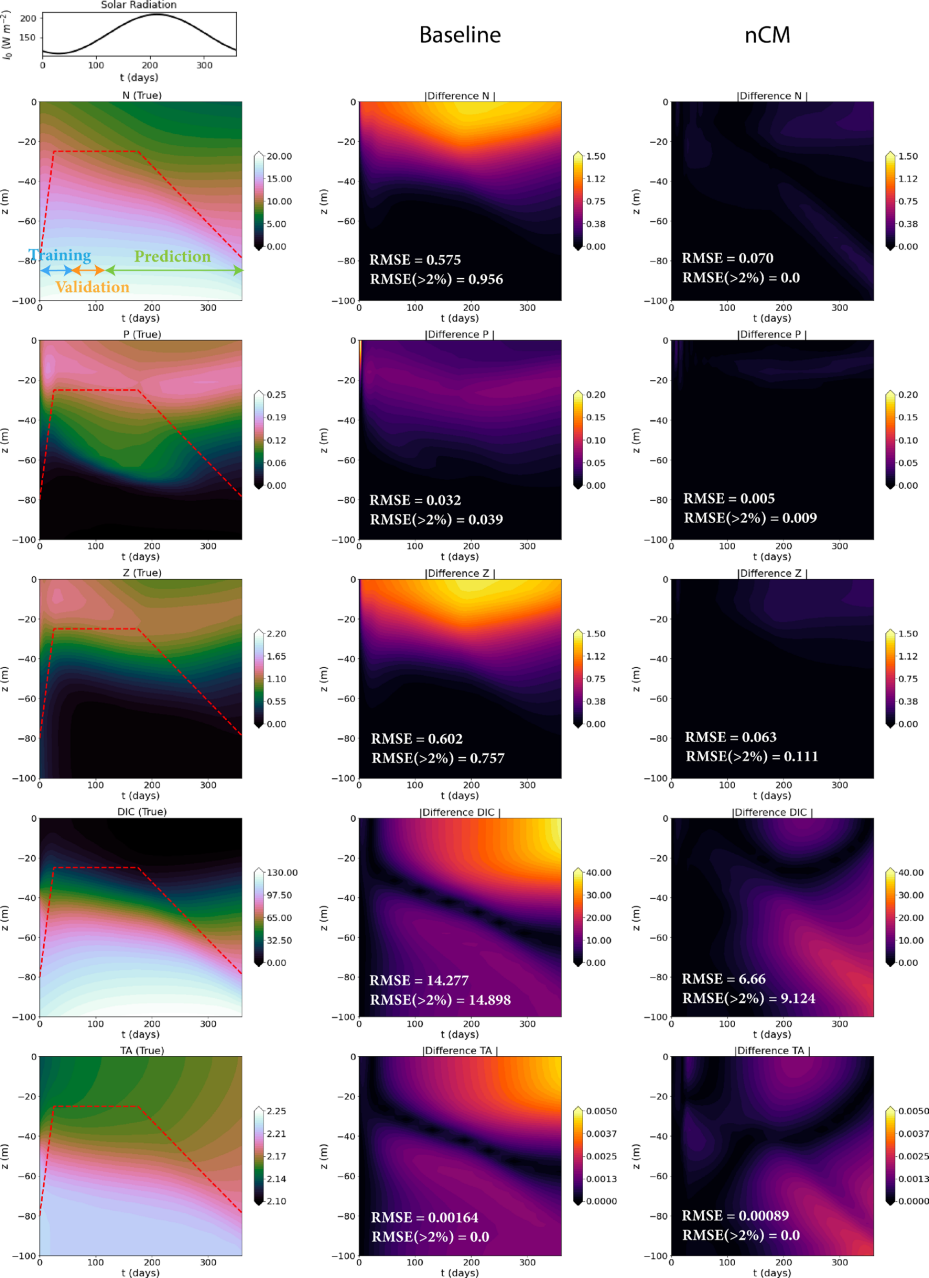
Comparison of the OA models used in Experiments-2b with and without closure models. The parameter values and concentration units are as in Fig. 6. For the *g*nCM, the training period is from t $=$ 0 to 60 days, the validation period from t $=$ 60 to 120 days, and the future prediction period from t $=$ 120 to 364 days. Left-column: the top panel shows the yearly variation of solar radiation and the subsequent panels depict the aggregated states from the NPZD-OA model with $M_Z(Z) = \frac{m_Z}{2}(Z + Z^2)$ (ground truth), overlaid with the dynamic mixed layer depth in dashed red lines. Middle-column: absolute difference between the corresponding states from the NPZ-OA model with $M_Z(Z) = \frac{m_Z}{2}Z$ (low-fidelity) and those in the left-column (high-fidelity ground truth). Right-column: absolute difference between the corresponding states from the low-fidelity model augmented with the learned *g*nCM and the ground truth. For each case, we also provide the pair of time-averaged errors (see Fig. 3 for description).

Numerics The numerical schemes used are as those of Experiments-2a. *Comparison LF-HF:* Since the high-fidelity NPZD-OA model resolves more processes, the concentrations of $N + D$ (aggregated state), *P*, *Z*, *DIC*, and *TA* differ significantly from the *N*, *P*, *Z*, *DIC*, and *TA* of the low-fidelity NPZ-OA model, as shown in Fig. 7.

*Training: NN architecture, data, and loss function* Our Markovian closure consists of a linear combination of a library of popular mortality functions^66^, $\{Z,Z^2, \frac{Z^2}{1+Z},\exp Z\}$. Once again, compared to the true zooplankton mortality term, our library contains three redundant terms, *Z*, $\frac{Z^2}{1+Z}$, and $\exp {Z}$, where the *Z* term is already a part of the low-fidelity model and completely known. Additionally, we use a deep-NN for the non-Markovian closure term, with *N*(*z*, *t*), *P*(*z*, *t*), *Z*(*z*, *t*), and *I*(*z*, *t*) as the input; the inclusion of the photosynthetically active radiation, *I*(*z*, *t*), makes this closure term non-autonomous. The architecture for the fully-connected deep-NN used in the non-Markovian closure term is provided in Table SI-1, and it consists of two hidden-layers with the non-linear *swish* activation. We do not include the states *DIC*(*z*, *t*) and *TA*(*z*, *t*) among the inputs in order to preserve one-way coupling between the biological and carbonate system. Along with this, biomass conservation and coupling of the carbonate system by nitrogen conversion (as in Eqs. 25 and 26) is maintained in the non-Markovian closure terms by manipulating the channels of the output layer. On the other hand, in the Markovian layer, these constraints are imposed by constraining the weights of the output layer. To help with learning, we further impose the condition that the contribution of the Markovian closure term to the *P* equation is exactly equal to zero. See Table SI-1 for implementational details of these constraints.

The training data consists of solving the NPZD-OA model with $M(Z) = \frac{m_Z}{2}(Z + Z^2)$, and the solution sampled at time intervals of 0.1 day until $t= 60~days$, $\{\{B^{true}(z, T_i)\}_{B\in \{N+D, P, Z, DIC, TA\}}\}_{i=1}^M$, i.e., $M=600$ high-fidelity solution states at different times. Performance of the learned model in the validation interval of 60 days $\le t \le$ 120 days is used to tune the hyperparameters, provided in Sect. SI-2.2. We again use a MAE based loss function, ${\mathcal {L}} = \frac{1}{M} \sum _{i=1}^M \int _0^{D} \frac{1}{D} \sqrt{\sum _{B \in \{N, P, Z, DIC, TA\}} \frac{1}{\sigma _{B}}|B^{pred}(z, T_i) - B^{true}(z, T_i)|} dz$, with $\sigma _N = 1, ~\sigma _{P} = 0.25, ~\sigma _Z = 1, ~\sigma _{DIC} = 2, ~\sigma _{TA} = 0.1$ (similar to those used in experiments-2a). A time delay of $\tau = 2.5~days$ was used for the non-Markovian closure term based on the optimal delay value study performed in^2^.

*Learning results* In nine repeats of the experiment with exactly the same set of hyperparameters, the mean and standard deviation of the learned contribution of the Markovian closure term to the *Z* equation is given by, $(-0.03000 \pm 0.00067) Z^2$. For reference, the true contribution of the quadratic mortality term to the *Z* equation is $-0.02998 Z^2$. Due to the weight constraints, the contribution of the Markovian closure term to other equations is exactly zero. We evaluate the performance of the learned neural closure model for long predictions, spanning over 1 year (365 *days*). The comparison with true/high-fidelity data for one of the experiments is provided in Fig. 7. Overall, the learned closure keeps the errors low throughout the 1-year time period, apart from a slight increase observed for the OA states after $\sim 200~days$.

*Sensitivity* Multiple experiments were done to study the effects of hyperparameters, such as batch-time, batch-size, regularization factors, etc., and their effects were similar to those observed in previous experiments. However, when using larger neural network architectures for the non-Markovian term, this led to high variability in the learned coefficients of the Markovian term on repeats of the experiments with the same set of hyperparameters. This is probably because of the increased expressive power of the non-Markovian term, which overshadows the significance of the learned Markovian term.

### Remarks and discussion

*Computational advantages* In^2^, through a flop-count analysis, we proved that the additional computational cost due to the presence of neural closure models is of similar or lower complexity than the existing low-fidelity model. However, in our current generalized framework, we have additional computational advantages. First, the size of the neural network architecture is completely independent of the number of discretized state variables and only dictated by the number of local features to be used as inputs to the *g*nCM terms. Second, as the same neural networks are applied locally at every grid point, it is directly possible to use batches of the size of the number of grid points. It has been reported that larger batch sizes could lead to performance speed-ups in forward pass through neural networks during the inference stage^67^. Estimating the leading flop-count order for training is non-trivial due to the presence of a number of operations ranging from time-integration of the forward model and adjoint PDEs; automatic differentiation through the neural networks; creation and use of interpolation functions; the integral to compute the final derivatives; the gradient descent step, etc. All these operations lead to training costs that are non-negligible. However, the generalizability and interpretability of our learned *g*nCMs over boundary conditions, initial conditions, domain, problem-specific parameters, etc., help justify the one-time training cost.

*Lack of prior knowledge* As showcased in the prior experiments and summarized in the corresponding sensitivity studies, the lack of prior knowledge about the missing dynamics could manifest in many different ways. This includes no known low-fidelity model, dynamics of the known low-fidelity model very different from the high-fidelity model/data, no knowledge of potential candidate terms to create input function libraries, or even no information on the most relevant state variables themselves. To allow compensation for this lack of prior knowledge, our *g*nCM framework is derived and implemented for any deep-neural-network (DNN) architectures for both Markovian and non-Markovian closure terms (Fig. 1). Our flexible modeling framework provides full autonomy for the design of the unknown closure terms such as using linear-, shallow-, or deep-NNs, selecting the span of the function libraries, and using either or both Markovian and non-Markovian closure terms. All these decisions are made by the subject matter expert/user depending on the problem at hand. For example, in all our experiments, fully-connected deep-NNs were utilized for the non-Markovian closure terms, because in general, no prior knowledge is available for the same. Further, our framework could be extended to allow for the adaptive increase of the input function library, for example using the algorithm proposed in^30^.

*Non-Markovian term* In the current derivation of the *g*nCM framework, due to the mathematical constructs, the non-Markovian term does not account for the possibility of memory decay contribution of the ${\mathcal {D}}_{NN}(\bullet )$ function under the integral w.r.t.  $t-s$ in Eq. (1). However, memory decay or other variations can be a desired property for some problems. To allow for this, one can split the integral in Eq. (1) into contiguous parts and multiply each of them with different weights. An alternate option is to consider discrete delays utilizing recurrent neural networks as for the discrete-nDDEs in^2^ and so implicitly incorporate the desired memory decay. In general, the need for the non-Markovian closure term for a given problem should be determined by the subject matter expert. However, in many cases, we anticipate the need for non-Markovian closure term to be imperative, especially when the high-fidelity model/data accounts for intermediate state variables not modeled in the known low-fidelity model, as in our “Experiments 2b: ocean acidification—model complexity”. Finally, it is also desirable to allow learning an adaptive optimal delay ($\tau$ in Eq. (1)), instead of treating it as a hyperparameter. For such a possibility, we refer to Appendix E in^68^ where we derive the theory for learning the optimal delay for the nCM framework (^2^; DDE counterpart for *g*nCM).

## Conclusions

In the present study, we develop neural closure models that are generalizable over computational grid resolution, boundary and initial conditions, domain geometries, and problem parameters, and also provide interpretability. These generalized neural closure models (*g*nCMs) are based on neural partial delay differential equations (nPDDEs) that augment existing/low-fidelity models in their PDE forms with both Markovian and non-Markovian closures parameterized with deep-NNs. The melding in the continuous spatiotemporal space is then followed by numerical discretization. This ensures that the burden of generalization, along with computing the relevant spatial derivatives, is carried by the numerical schemes, and not by the learned NNs. The space-time continuous form of the *g*nCMs also makes it easy to interpret the learned closures. For efficient training, we derive the adjoint PDEs in the continuous form and discretize them with adaptive time-integration schemes and employ interpolation functions constructed during forward integration to increase numerical stability and accuracy. This enables implementation across differentiable and non-differentiable computational physics codes, and different machine learning frameworks, all while being agnostic to the numerical methods. It further removes any requirements on the availability of regularly spaced training data in both space and time, and also accounts for errors in the time-evolution of the states in the presence of NNs during training. Finally, all our derivations and implementations consider deep-NN architectures for both Markovian and non-Markovian terms, thus automatically encompassing linear- and shallow-NNs, and providing the user or subject-matter-expert with the flexibility of choosing the architectural complexity in accord with prior knowledge.

Through a series of four sets of experiments, we demonstrate the interpretability and generalizability of our learned *g*nCMs. Our first two sets of simulation experiments are based on advecting nonlinear waves and shocks governed by the KdV-Burgers and classic Burgers PDEs, where the low-fidelity models are either missing terms or contain errors due to unresolved subgrid-scale processes. When presented with a function library containing terms of spatial derivatives of different orders and their combinations, grid-resolution, and the Reynolds number as inputs to the closure terms, our learned *g*nCMs eliminate redundant terms and discover missing physics, leading truncation error terms, and a correction to the nonlinear advection, all in an interpretable fashion. The correction to the nonlinear advection term, despite being absent from the input function library, is still accounted for and learned indirectly. Further, by analyzing the deep-NN weights, we also notice the learned closure terms to be independent of the Reynolds number. We find that training on data corresponding to just 3–4 combinations of a number of grid points and Reynolds number is sufficient to ensure that the learned closures are generalizable over large ranges of grid resolutions and Reynolds numbers, initial and boundary conditions, and also outperform the popular Smagorinsky closure model. Our last two sets of experiments are based on one-dimensional, non-autonomous ocean acidification PDE models, that couple physical, biological, and carbonate states, processes, and interactions. In these experiments, the low-fidelity models have uncertainty in the functional form of certain biological processes and lack complexity due to a missing intermediate state variable. The learned *g*nCMs simultaneously discriminate between candidate functional forms of the uncertain Zooplankton mortality term with the Markovian part of the closure, and account for the missing intermediate state and processes with the non-Markovian part. In terms of computational advantage, our new framework naturally lends itself to batching across computational grid points during the forward pass through the NNs in the closure terms, thus leading to potential performance speed-ups.

The *g*nCMs allow learning both Markovian and non-Markovian closure parameterization with deep-NNs at the PDE level, thus addressing the issues of generalizability and interpretability that are often the bottleneck when it comes to using machine learning for computational science and engineering problems. The generalizability and interpretability properties also make it easier to justify the often computationally expensive training stage, thus enabling wider adoption.

## Supplementary Information

Supplementary Information.

## Data Availability

The codes and data used in this work are available in the GitHub repository: https://github.com/mit-mseas/generalized_nCMs.git.
