# Supplementary material for: Generalized neural closure models with interpretability
Source: Sci Rep. 2023 Jun 30;13:10634. doi: 10.1038/s41598-023-35319-w (PMC10313723; doi:10.1038/s41598-023-35319-w)
Supplement: Supplementary file 1 — Supplementary Information. [file 41598_2023_35319_MOESM1_ESM.pdf]

Supplementary Information

Generalized Neural Closure Models with Interpretability

Abhinav Gupta*[1] and Pierre F.J. Lermusiaux†[1]

[1]Department of Mechanical Engineering, Massachusetts Institute of Technology, Cambridge, MA 02139

Saturday 25th March, 2023

SI-1 Adjoint Equations for Neural Partial Delay Differential Equations

In this section, we provide a detailed derivation of adjoint equations for neural partial delay differential equations (nPDDEs). This derivation is inspired by the adjoint equation derivation for a general PDE by Li and Petzold, 2004 [1] and Cao et al., 2002 [2]. Our nPDDE is of the form,

$$\begin{aligned}\frac{\partial u(x,t)}{\partial t} =&\mathcal{F}_{NN}\left(u(x,t), \frac{\partial u(x,t)}{\partial x}, \frac{\partial^2 u(x,t)}{\partial x^2}, ..., \frac{\partial^d u(x,t)}{\partial x^d}, x, t; \phi\right)\\ &+\int_{t-\tau}^{t} \mathcal{D}_{NN}\left(u(x,s), \frac{\partial u(x,s)}{\partial x}, \frac{\partial^2 u(x,s)}{\partial x^2}, ..., \frac{\partial^d u(x,s)}{\partial x^d}, x, s; \theta\right) ds\,, \qquad \text{(SI-1)}\\ &\qquad\qquad x\in\Omega,\ t\geq 0,\\ u(x,t) = h(x,t),&\ -\tau\leq t\leq 0 \quad \text{and} \quad \mathcal{B}(u(x,t)) = g(x,t),\ x\in\partial\Omega,\ t\geq 0\,.\end{aligned}$$

As compared to PDEs, PDDEs require the specification of a history function ($h(x,t)$) for the initial conditions. $\mathcal{F}_{NN}(\bullet;\phi)$ and $\mathcal{D}_{NN}(\bullet;\theta)$ are two neural networks (NNs) parameterized by ϕ and θ, respectively. We consider the presence of an arbitrary number of spatial derivatives, with the highest order defined by $d\in\mathbb{Z}^+$. We can rewrite the above equation SI-1 as an equivalent system

*guptaa@mit.edu

†pierrel@mit.edu

of coupled PDDEs with discrete delays,

$$
\begin{aligned}
\frac{\partial u(x,t)}{\partial t} =& \mathcal{F}_{NN}\left(u(x,t), \frac{\partial u(x,t)}{\partial x}, \frac{\partial^2 u(x,t)}{\partial x^2}, ..., \frac{\partial^d u(x,t)}{\partial x^d}, x, t; \phi\right) + y(x,t)\ , \\
& \qquad\qquad\qquad\qquad\qquad\qquad\qquad\qquad x \in \Omega,\ t \geq 0, \\
\frac{\partial y(x,t)}{\partial t} =& \mathcal{D}_{NN}\left(u(x,t), \frac{\partial u(x,t)}{\partial x}, \frac{\partial^2 u(x,t)}{\partial x^2}, ..., \frac{\partial^d u(x,t)}{\partial x^d}, x, t; \theta\right) \\
& - \mathcal{D}_{NN}\left(u(x,t-\tau), \frac{\partial u(x,t-\tau)}{\partial x}, \frac{\partial^2 u(x,t-\tau)}{\partial x^2}, ..., \frac{\partial^d u(x,t-\tau)}{\partial x^d}, x, t-\tau; \theta\right)\ , \\
& \qquad\qquad\qquad\qquad\qquad\qquad\qquad\qquad x \in \Omega,\ t \geq 0, \\
u(x,t) =& h(x,t), -\tau \leq t \leq 0 \quad \text{and} \quad \mathcal{B}(u(x,t)) = g(x,t), x \in \partial\Omega, t \geq 0 \\
y(x,0) =& \int_{-\tau}^{0} \mathcal{D}_{NN}\left(h(x,s), \frac{\partial h(x,s)}{\partial x}, \frac{\partial^2 h(x,s)}{\partial x^2}, ..., \frac{\partial^d h(x,s)}{\partial x^d} x, s; \theta\right) ds\,.
\end{aligned}
\tag{SI-2}
$$

Let us assume that high-fidelity data is available at M discrete times, $T_1 < ... < T_M \leq T$, and at $N(T_i)$ spatial locations ($x_k^{T_i} \in \Omega, \forall k \in 1, ..., N(T_i)$) for each of the times. We define the scalar loss function as $L = \frac{1}{M}\sum_{i=1}^{M} \frac{1}{N(T_i)} \sum_{k=1}^{N(T_i)} l(u(x_k^{T_i}, T_i)) \equiv \int_0^T \frac{1}{M}\sum_{i=1}^{M} \int_\Omega \frac{1}{N(T_i)} \sum_{k=1}^{N(T_i)} l(u(x,t))\delta(x - x_k^{T_i})\delta(t - T_i)dxdt \equiv \int_0^T \frac{1}{M}\sum_{i=1}^{M} \frac{1}{|\Omega|}\int_\Omega \hat{l}(u(x,t))\delta(t-T_i)dxdt$, where $l(\bullet)$ are scalar loss functions such as mean-squared-error (MSE), and $\delta(\bullet)$ is the Kronecker delta function. In order to derive the adjoint PDEs, we start with the Lagrangian corresponding to the above system,

$$
\begin{aligned}
\mathbb{L} =& L(u(x,t)) + \int_0^T \int_\Omega \lambda^T(x,t)\left(\partial_t u(x,t) - \mathcal{F}_{NN}(\bullet, t; \phi) - y(x,t)\right) dxdt \\
& + \int_0^T \int_\Omega \mu^T(x,t)\left(\partial_t y(x,t) - \mathcal{D}_{NN}(\bullet, t; \theta) + \mathcal{D}_{NN}(\bullet, t-\tau; \theta)\right) dxdt \\
& + \int_\Omega \alpha^T(x)\left(y(x,0) - \int_{-\tau}^{0} \mathcal{D}_{NN}(h(x,t), \partial_x h(x,t), \partial_{x^2} h(x,t), ..., \partial_{x^d} h(x,t), x, t; \theta) dt\right) dx\,,
\end{aligned}
\tag{SI-3}
$$

where $\lambda(x,t)$, $\mu(x,t)$ and $\alpha(x)$ are the Lagrangian variables. We then take the derivative of the Lagrangian (equation SI-3) w.r.t. θ (for brevity we denote, $\partial/\partial(\bullet) \equiv \partial_{(\bullet)}$, and $d/d(\bullet) \equiv d_{(\bullet)}$),

$$
\begin{aligned}
d_\theta \mathbb{L} =& \int_0^T \int_\Omega \frac{1}{M}\frac{1}{|\Omega|}\sum_{i=1}^{M} \partial_{u(x,t)} \hat{l}(u(x,t))\delta(t-T_i) d_\theta u(x,t) dxdt + \int_0^T \int_\Omega \lambda^T(x,t)\left(\partial_t d_\theta u(x,t)\right. \\
& - \partial_{u(x,t)} \mathcal{F}_{NN}(\bullet, t; \phi) d_\theta u(x,t) - \partial_{\partial_x u(x,t)} \mathcal{F}_{NN}(\bullet, t; \phi) d_\theta \partial_x u(x,t) \\
& \left.- \partial_{\partial_{xx} u(x,t)} \mathcal{F}_{NN}(\bullet, t; \phi) d_\theta \partial_{xx} u(x,t) - d_\theta y(x,t)\right) dxdt + \int_0^T \int_\Omega \mu^T(x,t)\left(\partial_t d_\theta y(x,t)\right. \\
& - \partial_{u(x,t)} \mathcal{D}_{NN}(\bullet, t; \theta) d_\theta u(x,t) - \partial_{\partial_x u(x,t)} \mathcal{D}_{NN}(\bullet, t; \theta) d_\theta \partial_x u(x,t) \\
& - \partial_{\partial_{xx} u(x,t)} \mathcal{D}_{NN}(\bullet, t; \theta) d_\theta \partial_{xx} u(x,t) \\
& - \partial_\theta \mathcal{D}_{NN}(\bullet, t; \theta) + \partial_{u(x,t)} \mathcal{D}_{NN}(\bullet, t-\tau; \theta) d_\theta u(x,t-\tau) \\
& + \partial_{\partial_x u(x,t-\tau)} \mathcal{D}_{NN}(\bullet, t-\tau; \theta) d_\theta \partial_x u(x,t-\tau) \\
& \left.+ \partial_{\partial_{xx} u(x,t-\tau)} \mathcal{D}_{NN}(\bullet, t-\tau; \theta) d_\theta \partial_{xx} u(x,t-\tau) + \partial_\theta \mathcal{D}_{NN}(\bullet, t-\tau; \theta)\right) dxdt \\
& + \int_\Omega \alpha^T(x)\left(d_\theta y(x,0) - \int_{-\tau}^{0} \partial_\theta G_{NN}(h(x,t), \partial_x h(x,t), \partial_{xx} h(x,t), x, t; \theta) dt\right) dx\,.
\end{aligned}
\tag{SI-4}
$$

Using integration-by-parts, we get,

$$\int_0^T \int_\Omega \lambda^T(x,t)\partial_t d_\theta u(x,t) dxdt = \int_\Omega [\lambda^T(x,t) d_\theta u(x,t)]\Big|_0^T - \int_0^T \int_\Omega \partial_t \lambda^T(x,t) d_\theta u(x,t) dxdt\,, \tag{SI-5}$$

$$\begin{aligned} &\int_0^T \int_\Omega \lambda^T(x,t)\partial_{\partial_x u(x,t)}\mathcal{F}_{NN}(\bullet,t)\partial_x d_\theta u(x,t) dxdt \\ &\qquad = \int_0^T \int_{\partial\Omega} \lambda^T(x,t)\partial_{\partial_x u(x,t)}\mathcal{F}_{NN}(\bullet,t) d_\theta u(x,t) dxdt \\ &\qquad\quad - \int_0^T \int_\Omega \partial_x \left(\lambda^T(x,t)\partial_{\partial_x u(x,t)}\mathcal{F}_{NN}(\bullet,t)\right) d_\theta u(x,t) dxdt\,, \end{aligned} \tag{SI-6}$$

$$\begin{aligned} &\int_0^T \int_\Omega \lambda^T(x,t)\partial_{\partial_{xx} u(x,t)}\mathcal{F}_{NN}(\bullet,t)\partial_{xx} d_\theta u(x,t) dxdt \\ &\qquad = \int_0^T \int_{\partial\Omega} \lambda^T(x,t)\partial_{\partial_{xx} u(x,t)}\mathcal{F}_{NN}(\bullet,t)\partial_x d_\theta u(x,t) dxdt \\ &\qquad\quad - \int_0^T \int_\Omega \partial_x \left(\lambda^T(x,t)\partial_{\partial_{xx} u(x,t)}\mathcal{F}_{NN}(\bullet,t)\right) \partial_x d_\theta u(x,t) dxdt \\ &\qquad = \int_0^T \int_{\partial\Omega} \lambda^T(x,t)\partial_{\partial_{xx} u(x,t)}\mathcal{F}_{NN}(\bullet,t)\partial_x d_\theta u(x,t) dxdt \\ &\qquad\quad - \left(\int_0^T \int_{\partial\Omega} \partial_x \left(\lambda^T(x,t)\partial_{\partial_{xx} u(x,t)}\mathcal{F}_{NN}(\bullet,t)\right) d_\theta u(x,t) dxdt \right.\\ &\qquad\quad \left. - \int_0^T \int_\Omega \partial_{xx} \left(\lambda^T(x,t)\partial_{\partial_{xx} u(x,t)}\mathcal{F}_{NN}(\bullet,t)\right) d_\theta u(x,t) dxdt\right)\,, \end{aligned} \tag{SI-7}$$

$$\begin{aligned} &\int_0^T \int_\Omega \mu^T(x,t)\partial_{u(x,t-\tau)}\mathcal{D}_{NN}(\bullet,t-\tau) d_\theta u(x,t-\tau) dxdt \\ &\qquad = \int_{-\tau}^{T-\tau} \int_\Omega \mu^T(x,t+\tau)\partial_{u(x,t)}\mathcal{D}_{NN}(\bullet,t) d_\theta u(x,t) dxdt \\ &\qquad = \int_0^T \int_\Omega \mu^T(x,t+\tau)\partial_{u(x,t)}\mathcal{D}_{NN}(\bullet,t) d_\theta u(x,t) dxdt \\ &\qquad\quad - \int_{T-\tau}^T \int_\Omega \mu^T(x,t+\tau)\partial_{u(x,t)}\mathcal{D}_{NN}(\bullet,t) d_\theta u(x,t) dxdt \\ &\qquad\quad + \int_{-\tau}^0 \int_\Omega \mu^T(x,t+\tau)\partial_{u(x,t)}\mathcal{D}_{NN}(\bullet,t) d_\theta u(x,t) dxdt\,. \end{aligned} \tag{SI-8}$$

Using the fact that $\mu^T(x,t) = 0, \forall t \geq T$ and $d_\theta u(x,t) = 0, -\tau \leq t \leq 0$, we get,

$$\begin{aligned} &\int_0^T \int_\Omega \mu^T(x,t)\partial_{u(x,t-\tau)}\mathcal{D}_{NN}(\bullet,t-\tau;\theta) d_\theta u(x,t-\tau) dxdt \\ &\qquad = \int_0^T \int_\Omega \mu^T(x,t+\tau)\partial_{u(x,t)}\mathcal{D}_{NN}(\bullet,t;\theta) d_\theta u(x,t) dxdt\,. \end{aligned} \tag{SI-9}$$

Similarly, we also get,

$$\begin{aligned}\int_0^T \int_\Omega \mu^T(x,t)\partial_{\partial_x u(x,t-\tau)}\mathcal{D}_{NN}(\bullet, t-\tau;\theta)\partial_x d_\theta u(x,t-\tau)dxdt \\ = \int_0^T \int_{\partial\Omega} \mu^T(x,t+\tau)\partial_{\partial_x u(x,t)}\mathcal{D}_{NN}(\bullet,t;\theta)d_\theta u(x,t)dxdt \\ - \int_0^T \int_\Omega \partial_x \left(\mu^T(x,t+\tau)\partial_{\partial_x u(x,t)}\mathcal{D}_{NN}(\bullet,t;\theta)\right) d_\theta u(x,t)dxdt\,,\end{aligned} \tag{SI-10}$$

$$\begin{aligned}\int_0^T \int_\Omega \mu^T(x,t)\partial_{\partial_{xx} u(x,t-\tau)}\mathcal{D}_{NN}(\bullet, t-\tau;\theta)\partial_{xx} d_\theta u(x,t-\tau)dxdt \\ = \int_0^T \int_{\partial\Omega} \mu^T(x,t+\tau)\partial_{\partial_{xx} u(x,t)}\mathcal{D}_{NN}(\bullet,t;\theta)\partial_x d_\theta u(x,t)dxdt \\ - \left(\int_0^T \int_{\partial\Omega} \partial_x \left(\mu^T(x,t+\tau)\partial_{\partial_{xx} u(x,t)}\mathcal{D}_{NN}(\bullet,t;\theta)\right) d_\theta u(x,t)dxdt \right.\\ \left. - \int_0^T \int_\Omega \partial_{xx} \left(\mu^T(x,t+\tau)\partial_{\partial_{xx} u(x,t)}\mathcal{D}_{NN}(\bullet,t;\theta)\right) d_\theta u(x,t)dxdt \right)\,.\end{aligned} \tag{SI-11}$$

Plugging everything in yields,

$$\begin{aligned} d_\theta \mathbb{L} = & \int_0^T \int_\Omega \frac{1}{M}\frac{1}{|\Omega|}\sum_{i=1}^{M} \partial_{u(x,t)}\hat{l}(u(x,t))\delta(t-T_i)d_\theta u(x,t)dxdt \\ & - \int_0^T \int_\Omega \partial_t \lambda^T(x,t)d_\theta u(x,t)dxdt \\ & - \int_0^T \int_\Omega \lambda^T(x,t)\partial_{u(x,t)}\mathcal{F}_{NN}(\bullet,t)d_\theta u(x,t)dxdt \\ & - \int_0^T \int_{\partial\Omega} \lambda^T(x,t)\partial_{\partial_x u(x,t)}\mathcal{F}_{NN}(\bullet,t)d_\theta u(x,t)dxdt \\ & + \int_0^T \int_\Omega \partial_x \left(\lambda^T(x,t)\partial_{\partial_x u(x,t)}\mathcal{F}_{NN}(\bullet,t)\right) d_\theta u(x,t)dxdt \\ & - \int_0^T \int_{\partial\Omega} \lambda^T(x,t)\partial_{\partial_{xx} u(x,t)}\mathcal{F}_{NN}(\bullet,t)\partial_x d_\theta u(x,t)dxdt \\ & + \int_0^T \int_{\partial\Omega} \partial_x \left(\lambda^T(x,t)\partial_{\partial_{xx} u(x,t)}\mathcal{F}_{NN}(\bullet,t)\right) d_\theta u(x,t)dxdt \\ & - \int_0^T \int_\Omega \partial_{xx} \left(\lambda^T(x,t)\partial_{\partial_{xx} u(x,t)}\mathcal{F}_{NN}(\bullet,t)\right) d_\theta u(x,t)dxdt \\ & - \int_0^T \int_\Omega \lambda^T(x,t)d_\theta y(x,t)dxdt \\ & - \int_\Omega \mu^T(x,0)d_\theta y(x,0)dx - \int_0^T \int_\Omega \partial_t \mu^T(x,t)d_\theta y(x,t)dxdt \\ & - \int_0^T \int_\Omega \mu^T(x,t)\partial_{u(x,t)}\mathcal{D}_{NN}(\bullet,t;\theta)d_\theta u(x,t)dxdt \\ & - \int_0^T \int_{\partial\Omega} \mu^T(x,t)\partial_{\partial_x u(x,t)}\mathcal{D}_{NN}(\bullet,t;\theta)d_\theta u(x,t)dxdt \end{aligned}$$

$$
\begin{aligned}
&+ \int_0^T \int_\Omega \partial_x \left(\mu^T(x,t)\partial_{\partial_x u(x,t)}\mathcal{D}_{NN}(\bullet,t;\theta)\right) d_\theta u(x,t) dx dt \\
&- \int_0^T \int_{\partial\Omega} \mu^T(x,t)\partial_{\partial_{xx} u(x,t)}\mathcal{D}_{NN}(\bullet,t;\theta)\partial_x d_\theta u(x,t) dx dt \\
&+ \int_0^T \int_{\partial\Omega} \partial_x \left(\mu^T(x,t)\partial_{\partial_{xx} u(x,t)}\mathcal{D}_{NN}(\bullet,t;\theta)\right) d_\theta u(x,t) dx dt \\
&- \int_0^T \int_\Omega \partial_{xx} \left(\mu^T(x,t)\partial_{\partial_{xx} u(x,t)}\mathcal{D}_{NN}(\bullet,t;\theta)\right) d_\theta u(x,t) dx dt \\
&- \int_0^T \int_\Omega \mu^T(x,t)\partial_\theta \mathcal{D}_{NN}(\bullet,t;\theta) dx dt \\
&+ \int_0^T \int_\Omega \mu^T(x,t+\tau)\partial_{u(x,t)}\mathcal{D}_{NN}(\bullet,t;\theta) d_\theta u(x,t) dx dt \\
&+ \int_0^T \int_{\partial\Omega} \mu^T(x,t+\tau)\partial_{\partial_x u(x,t)}\mathcal{D}_{NN}(\bullet,t;\theta) d_\theta u(x,t) dx dt \\
&- \int_0^T \int_\Omega \partial_x \left(\mu^T(x,t+\tau)\partial_{\partial_x u(x,t)}\mathcal{D}_{NN}(\bullet,t;\theta)\right) d_\theta u(x,t) dx dt \\
&+ \int_0^T \int_{\partial\Omega} \mu^T(x,t+\tau)\partial_{\partial_{xx} u(x,t)}\mathcal{D}_{NN}(\bullet,t;\theta)\partial_x d_\theta u(x,t) dx dt \\
&- \int_0^T \int_{\partial\Omega} \partial_x \left(\mu^T(x,t+\tau)\partial_{\partial_{xx} u(x,t)}\mathcal{D}_{NN}(\bullet,t;\theta)\right) d_\theta u(x,t) dx dt \\
&+ \int_0^T \int_\Omega \partial_{xx} \left(\mu^T(x,t+\tau)\partial_{\partial_{xx} u(x,t)}\mathcal{D}_{NN}(\bullet,t;\theta)\right) d_\theta u(x,t) dx dt \\
&+ \int_0^T \int_\Omega \mu^T(x,t)\partial_\theta \mathcal{D}_{NN}(\bullet,t-\tau;\theta) dx dt \\
&+ \int_\Omega \alpha^T(x) d_\theta y(x,0) \\
&- \int_\Omega \alpha^T(x) \int_{-\tau}^0 \partial_\theta \mathcal{D}_{NN}(h(x,t),\partial_x h(x,t),\partial_{xx} h(x,t),x,t;\theta) dt dx \,. \qquad \text{(SI-12)}
\end{aligned}
$$

Collecting all the terms with $\int_\Omega$, $d_\theta u(x,t)$, and $d_\theta y(x,t)$, we get the following adjoint PDEs,

$$
\begin{aligned}
0 = {} & \frac{1}{M}\frac{1}{|\Omega|}\sum_{k=1}^{M} \partial_{u(x,t)}\hat{l}(u(x,t))\delta(t-T_k) \\
& - \partial_t \lambda^T(x,t) - \lambda^T(x,t)\partial_{u(x,t)}\mathcal{F}_{NN}(\bullet,t) + \sum_{i=1}^{d}(-1)^{i+1}\partial_{x^i}\left(\lambda^T(x,t)\partial_{\partial_{x^i} u(x,t)}\mathcal{F}_{NN}(\bullet,t)\right) \\
& - \mu^T(x,t)\partial_{u(x,t)}\mathcal{D}_{NN}(\bullet,t;\theta) + \sum_{i=1}^{d}(-1)^{i+1}\partial_{x^i}\left(\mu^T(x,t)\partial_{\partial_{x^i} u(x,t)}\mathcal{D}_{NN}(\bullet,t;\theta)\right) \qquad \text{(SI-13)} \\
& + \mu^T(x,t+\tau)\partial_{u(x,t)}\mathcal{D}_{NN}(\bullet,t;\theta) - \sum_{i=1}^{d}(-1)^{i+1}\partial_{x^i}\left(\mu^T(x,t+\tau)\partial_{\partial_{x^i} u(x,t)}\mathcal{D}_{NN}(\bullet,t;\theta)\right)\,, \\
& \qquad x \in \Omega\,,\ t \in [0,T)\,, \\
0 = {} & -\lambda^T(x,t) - \partial_t \mu^T(x,t)\,, \quad x \in \Omega\,,\ t \in [0,T)\,,
\end{aligned}
$$

with initial conditions, $\lambda(x,t) = \mu(x,t) = 0,\ t \geq T$. The boundary conditions are derived based on that of the forward PDE such that they satisfy,

$$
\begin{aligned}
0 = &\sum_{i=0}^{d} \sum_{j=0}^{d-i-1} (-1)^{j+1} \partial_{x^j} \left(\lambda^T(x,t) \partial_{\partial_{x^{j+i+1}} u(x,t)} \mathcal{F}_{NN}(\bullet, t) \right) d_\theta \partial_{x^i} u(x,t) \\
+ &\sum_{i=0}^{d} \sum_{j=0}^{d-i-1} (-1)^{j+1} \partial_{x^j} \left(\mu^T(x,t) \partial_{\partial_{x^{j+i+1}} u(x,t)} \mathcal{D}_{NN}(\bullet, t) \right) d_\theta \partial_{x^i} u(x,t) \\
- &\sum_{i=0}^{d} \sum_{j=0}^{d-i-1} (-1)^{j+1} \partial_{x^j} \left(\mu^T(x,t+\tau) \partial_{\partial_{x^{j+i+1}} u(x,t)} \mathcal{D}_{NN}(\bullet, t) \right) d_\theta \partial_{x^i} u(x,t)\ , \\
&\qquad x \in \partial\Omega,\ t \in [t, T)\,.
\end{aligned} \tag{SI-14}
$$

Note that adjoint PDE needs to be solved backward in time, and one would require access to $u(x,t), \forall x \in \Omega,\ 0 \leq t \leq T$. After solving for the Lagrangian variables, $\lambda(x,t)$ and $\mu(x,t)$, we can compute the required gradients as follows:

$$
\begin{aligned}
d_\theta \mathcal{L} = &- \int_0^T \int_\Omega \mu^T(x,t) \partial_\theta \mathcal{D}_{NN}(\bullet, t; \theta) dx dt + \int_0^T \int_\Omega \mu^T(x,t) \partial_\theta \mathcal{D}_{NN}(\bullet, t - \tau; \theta) dx dt \\
&- \int_\Omega \mu^T(x,0) \int_{-\tau}^0 \partial_\theta \mathcal{D}_{NN}(h(x,t), \partial_x h(x,t), \partial_{xx} h(x,t), x, t; \theta) dt dx\,.
\end{aligned} \tag{SI-15}
$$

If we restart the above derivation by taking derivative of the Lagrangian (equation SI-3) w.r.t. ϕ, we will arrive at the same adjoint PDEs (equations SI-13 & SI-14), and the required gradient will be given by,

$$
d_\phi \mathcal{L} = - \int_0^T \int_\Omega \lambda^T(x,t) \partial_\phi \mathcal{F}_{NN}(\bullet, t; \phi) dx dt\,. \tag{SI-16}
$$

Finally, using any stochastic gradient descent algorithm, we can find the optimal values of the weights ϕ and θ.

SI-2 Experimental Setup

SI-2.1 Architectures

The architectures used to generate the results corresponding to different experiments are provided in table SI-1. The implementation details of the various biological and carbonate constraints imposed on the neural closure terms in experiments 2a & 2b are also provided.

SI-2.2 Hyperparameters

The values of the tuned training hyperparameters corresponding to different experiments are listed next. In all the experiments, the number of iterations per epoch is calculated by dividing the number of time-steps in the training period by the batch-size multiplied by the length of short time-sequences, adding 1, and rounding up to the next integer.

SI-2.2.1 Experiments-1a:

For training, we randomly select short time-sequences spanning 3 time-steps (batch-time) and extract data at every time-step to form batches of size 16; 4 iterations per epoch are used; an exponentially decaying learning rate (LR) schedule is used with an initial LR of 0.075, the decay rate of 0.97, and 4 decay steps; the RMSprop optimizer is employed; training is for a total of 150 epochs. $\mathcal{L}_1$ and $\mathcal{L}_2$ regularization with factors of 1.5×10^{-3} and 1×10^{-5}, respectively, is used; the weights are pruned if the value drops below 5×10^{-3}.

SI-2.2.2 Experiments-1b:

Only Markovian closure case: We randomly select short time-sequences spanning 30 time-steps (batch-time) and extract data at every other time-step to form batches of size 2. In total 24 iterations per epoch are used, with every 8 of them belonging to one of the (N_x, Re) pairs. An exponentially decaying learning rate (LR) schedule with an initial LR of 0.025, a decay rate of 0.95, and 24 decay steps are used; the RMSprop optimizer is used; we train for a total of 30 epochs. We also use both $\mathcal{L}_1$ and $\mathcal{L}_2$ regularization for the weights of the neural network with factors of 5×10^{-4} and 5×10^{-4}, respectively, along with pruning of the weights if their value drops below 5×10^{-3}.
Both Markovian and non-Markovian closures case: A batch-time of 30 time-steps is used with data extracted at every other time-step to form batches of size 2; 32 iterations per epoch are used, with every 8 of them belonging to one of the (N_x, Re) pairs; an exponentially decaying learning rate (LR) schedule is employed with an initial LR of 0.01, the decay rate of 0.95, and 32 decay steps; the RMSprop optimizer is used; we train for a total of 30 epochs. We also use both $\mathcal{L}_1$ and $\mathcal{L}_2$ regularization for the weights of the neural network with factors of 1.5×10^{-3} and 1×10^{-5}, respectively, along with pruning of the weights if their value drops below 5×10^{-3}.

SI-2.2.3 Experiments-2a:

Parameter values used in the ocean acidification model are (adopted from [57; 3]): $g_{max} = 0.7\, day^{-1}$; $k_W = 0.08\, m^{-1}$; $K_N = 0.5\, mmol\ N\ m^{-3}$; $K_P = 0.25\, mmol\ N\ m^{-3}$; $m_P = 0.08\, day^{-1}(mmol\ N\ m^{-3})^{-1}$; $m_Z = 0.030\ day^{-1}(mmol\ N\ m^{-3})^{-1}$; $\mu_{max} = 2.808\ day^{-1}$; $\alpha = 0.14\ (W\ m^{-2}\ day)^{-1}$; $\beta = 0.0028\ (W\ m^{-2}\ day)^{-1}$; $\epsilon = 0.015\ day^{-1}$; $\lambda = 0.3$; $\gamma = 0.4$; a sinusoidal variation in $I_o(t)$; linear vertical variation in total biomass $T_{bio}(z)$ from 10 $mmol\ N\ m^{-3}$ at the surface to 20 $mmol\ N\ m^{-3}$ at $z = 100\ m$; $K_{z_b} = 0.0864\ (m^2/day)$; $K_{z_0} = 8.64\ (m^2/day)$; $\gamma_t = 0.1\ m^{-1}$; $D_z = -100\ m$; and $\rho_w = 1000\ kg/m^3$. For training, we randomly select short time-sequences spanning 3 time-steps (batch-time) and extract data at every other time-step to form batches of size 4; we use 26 iterations per epoch; an exponentially decaying learning rate (LR) schedule is used with an initial LR of 0.075, the decay rate of 0.97, and 26 decay steps; the RMSprop optimizer is adopted; training is terminated at 200 epochs. We also use both $\mathcal{L}_1$ and $\mathcal{L}_2$ regularization for the weights of the neural network with factors of 1.5×10^{-3} and 1×10^{-3}, respectively, along with pruning of the weights if their value drops below 5×10^{-3}.

SI-2.2.4 Experiments-2b:

We use a batch-time of 3 time-steps with data extracted at every other time-step to form batches of size 8; we use 26 iterations per epoch; an exponentially decaying learning rate (LR) schedule is applied with an initial LR of 0.075, the decay rate of 0.97, and 26 decay steps; the RMSprop optimizer is employed; training is terminated at 200 epochs. We also use both $\mathcal{L}_1$ and $\mathcal{L}_2$ regularization

for the weights of the Markovian closure with factors of 1.5×10^{-3} and 1×10^{-3}, respectively, along with pruning of the weights if their value drops below 5×10^{-3}. For the neural network in the non-Markovian closure term, only $\mathcal{L}_2$ regularization with a factor of 1×10^{-5} is used.

Finally, for all the experiments and their multiple repeats with the exact same tuned hyper-parameters, we provide variation of training and validation error as training progresses (figure SI-1).

References

[1] Shengtai Li and Linda Petzold. Adjoint sensitivity analysis for time-dependent partial differential equations with adaptive mesh refinement. *Journal of Computational Physics*, 198(1): 310–325, 2004.

[2] Yang Cao, Shengtai Li, and Linda Petzold. Adjoint sensitivity analysis for differential-algebraic equations: algorithms and software. *Journal of computational and applied mathematics*, 149(1): 171–191, 2002.

[3] Mette Eknes and Geir Evensen. An ensemble kalman filter with a 1-d marine ecosystem model. *Journal of Marine Systems*, 36(1-2):75–100, 2002.

Experiments	Markovian Term			Non-Markovian Term			
	**Architecture**	**Act.**	**Trainable Weights**	**Architecture**	**Act.**	**Delays**	**Trainable Weights**
1a	$\mathcal{F}_{NN}$		4				
	Input layer with 4 neurons	none					
	Dense output layer with 1 neurons	linear					
1b	$\mathcal{F}_{NN}$		4	$\mathcal{G}_{NN}$		0.075	198
	Input layer with 4 neurons	none		Input layer with 5 neurons	none		
	Dense output layer with 1 neurons	linear		Dense hidden layer with 10 neurons	swish		
				Dense hidden layer with 7 neurons	swish		
				2 Dense hidden layers with 5 neurons	swish		
				Dense hidden layer with 3 neurons	swish		
				Dense output layer with 1 neuron	linear		
				Multiply output with $\lvert u \rvert$			
2a	$\mathcal{F}_{NN}$		18 (effective)<br>$w = \begin{bmatrix} -(w_2 + w_3 + w_4) \\ w_2 \\ w_3 \\ w_4 \\ -C_P w_2 - C_Z w_3 - C_D w_4 \\ (w_2 + w_3 + w_4)/\rho_w \end{bmatrix}$				
	Input layer with 6 neurons	none					
	Dense output layer with 6 neurons	linear					
2b	$\mathcal{F}_{NN}$		4 (effective)<br>$w = \begin{bmatrix} -w_3 \\ 0 \\ w_3 \\ -C_Z w_3 \\ w_3/\rho_w \end{bmatrix}$	$\mathcal{G}_{NN}$		2.5	65
	Input layer with 4 neurons	none		Input layer with 4 neurons	none		
	Dense output layer with 5 neurons	linear		2 Dense hidden layer with 5 neurons	swish		
				Dense output layer with 4 neurons	linear		
				$\mathcal{G}_{NN} = \begin{bmatrix} -(\mathcal{G}^1_{NN} + \mathcal{G}^2_{NN}) \\ \mathcal{G}^1_{NN} \\ \mathcal{G}^2_{NN} \\ \mathcal{G}^3_{NN} \\ \mathcal{G}^4_{NN}/\rho_w \end{bmatrix}$			

Table SI-1: Architectures for different generalized neural closure models used in the four sets of experiments. We explicitly provide the constraints on the weights and output layer of neural networks used in different experiments. $\{w_i\}_{i=1}^4$ are row vectors of the weight matrix. "Effective" number of trainable weights does not count the ones which are not free or are overwritten due to the imposed constraints. C_P, C_Z, and C_D are the carbon-nitrogen ratios for phytoplanktons, zooplanktons, and detritus, respectively. ρ_w is seawater density.

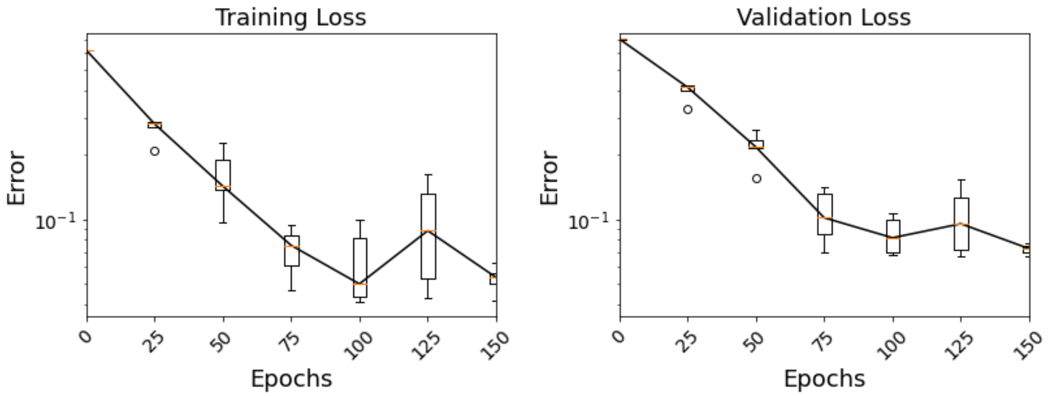

(a) Experiments-1a

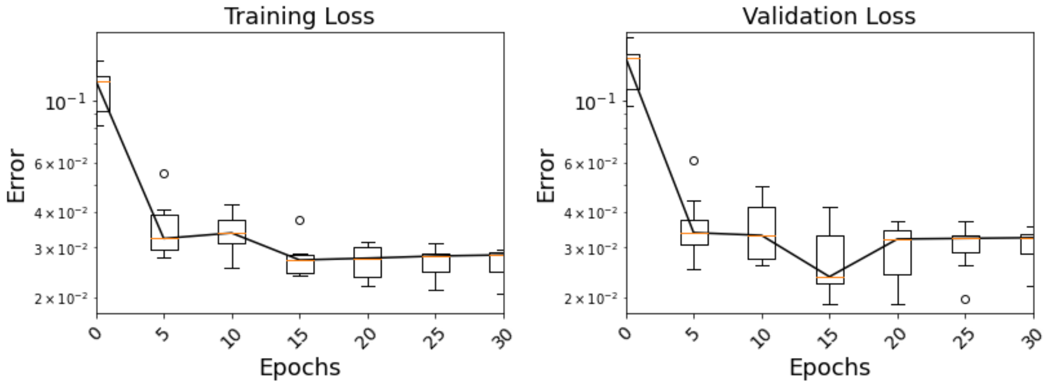

(b) Experiments-1b (gnCM with only Markovian closure term)

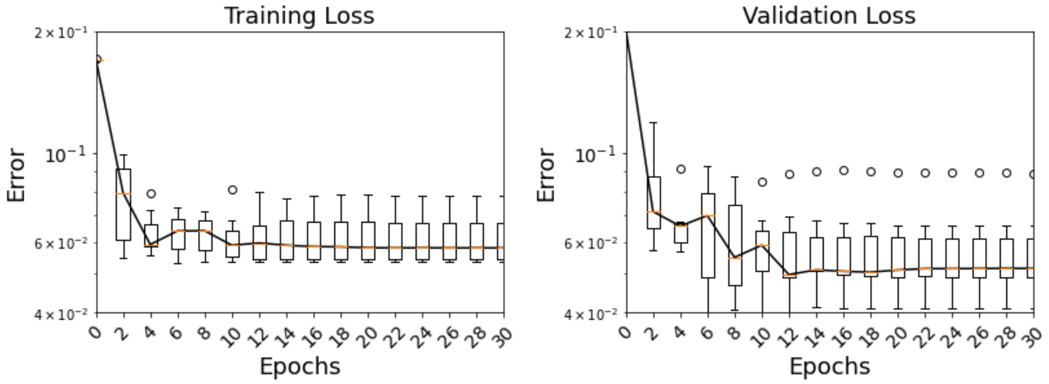

(c) Experiments-1b (gnCM with both Markovian and non-Markovian closure terms)

Figure SI-1: Variation of training (left column) and validation (right column) loss with epochs, for each of the experiments-1a, 1b, 2a, and 2b. We use boxplots to provide statistical summaries for multiple training repeats done for each set of experiments. The box and its whiskers provide a five-number summary: minimum, first quartile (Q1), median (orange solid line), third quartile (Q3), and maximum, along with outliers (black circles) if any. These results accompany the architectures detailed in table SI-1. *(cont.)*

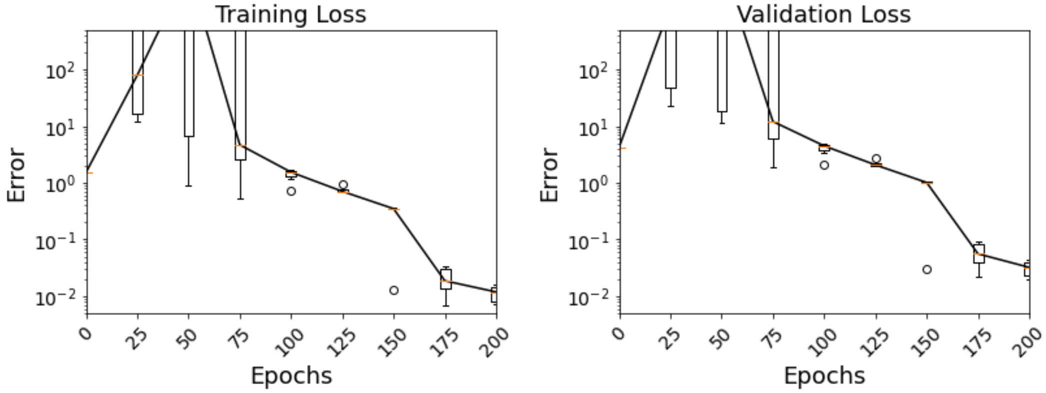

(d) Experiments-2a

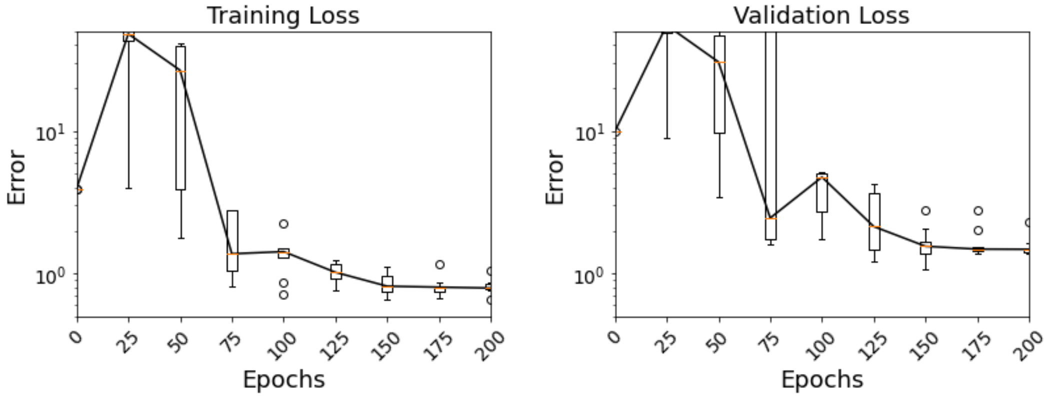

(e) Experiments-2b

Figure SI-1: Variation of training (left column) and validation (right column) loss with epochs, for each of the experiments-1a, 1b, 2a, and 2b. We use boxplots to provide statistical summaries for multiple training repeats done for each set of experiments. The box and its whiskers provide a five-number summary: minimum, first quartile (Q1), median (orange solid line), third quartile (Q3), and maximum, along with outliers (black circles) if any. These results accompany the architectures detailed in table SI-1.
